# Autonomous assembly of synthetic oligonucleotides built from an expanded DNA alphabet. Total synthesis of a gene encoding kanamycin resistance

**DOI:** 10.3762/bjoc.10.245

**Published:** 2014-10-09

**Authors:** Kristen K Merritt, Kevin M Bradley, Daniel Hutter, Mariko F Matsuura, Diane J Rowold, Steven A Benner

**Affiliations:** 1 Foundation for Applied Molecular Evolution, P.O. Box 13174, Gainesville, FL, 32604, USA; 2 The Westheimer Institute for Science and Technology, 720 S. W. 2nd Avenue, Suites 201-208, Gainesville, FL, 32601, USA; 3 Firebird Biomolecular Sciences LLC, 13709 Progress Blvd. Box 17, Alachua, FL 32615, USA; 4 Department of Chemistry, University of Florida, Gainesville, FL, 32611, USA

**Keywords:** automated gene synthesis, artificially expanded genetic information systems, solid-phase DNA synthesis, synthetic biology

## Abstract

**Background:** Many synthetic biologists seek to increase the degree of autonomy in the assembly of long DNA (L-DNA) constructs from short synthetic DNA fragments, which are today quite inexpensive because of automated solid-phase synthesis. However, the low information density of DNA built from just four nucleotide “letters”, the presence of strong (G:C) and weak (A:T) nucleobase pairs, the non-canonical folded structures that compete with Watson–Crick pairing, and other features intrinsic to natural DNA, generally prevent the autonomous assembly of short single-stranded oligonucleotides greater than a dozen or so.

**Results:** We describe a new strategy to autonomously assemble L-DNA constructs from fragments of synthetic single-stranded DNA. This strategy uses an artificially expanded genetic information system (AEGIS) that adds nucleotides to the four (G, A, C, and T) found in standard DNA by shuffling hydrogen-bonding units on the nucleobases, all while retaining the overall Watson–Crick base-pairing geometry. The added information density allows larger numbers of synthetic fragments to self-assemble without off-target hybridization, hairpin formation, and non-canonical folding interactions. The AEGIS pairs are then converted into standard pairs to produce a fully natural L-DNA product. Here, we report the autonomous assembly of a gene encoding kanamycin resistance using this strategy. Synthetic fragments were built from a six-letter alphabet having two AEGIS components, 5-methyl-2’-deoxyisocytidine and 2’-deoxyisoguanosine (respectively **S** and **B**), at their overlapping ends. Gaps in the overlapped assembly were then filled in using DNA polymerases, and the nicks were sealed by ligase. The **S**:**B** pairs in the ligated construct were then converted to T:A pairs during PCR amplification. When cloned into a plasmid, the product was shown to make *Escherichia coli* resistant to kanamycin. A parallel study that attempted to assemble similarly sized genes with optimally designed standard nucleotides lacking AEGIS components gave successful assemblies of up to 16 fragments, but generally failed when larger autonomous assemblies were attempted.

**Conclusion:** AEGIS nucleotides, by increasing the information density of DNA, allow larger numbers of DNA fragments to autonomously self-assemble into large DNA constructs. This technology can therefore increase the size of DNA constructs that might be used in synthetic biology.

## Introduction

It has been nearly 50 years since the first solid-phase synthesis of DNA by Letsinger and Mahadevan [1–2]. This work laid the platform for new strategies in oligonucleotide synthesis, culminating in the development of phosphoramidite-based synthesis of DNA in the 1980s [3]. The later full automation of these processes has caused many non-chemists to take, almost for granted, the low cost of synthetic DNA fragments. For example, when announcing the Defense Advanced Research Projects Agency’s (DARPA) 2011 initiative in “Living Foundries,” the program manager is quoted as saying that “DNA should cost next to nothing” [4]. Indeed, the DARPA Foundries 1000 molecules program hopes for the assembly of large DNA (L-DNA) “chassis” via fully automated processes.

Historically, essentially all syntheses of L-DNA constructs, including synthesis of complete genes, have followed a convergent assembly strategy. This was used for the first time for the total synthesis of genes encoding transfer RNA [5], but convergent synthesis is generally routine for the synthesis of natural products [6]. In the convergent synthesis of L-DNA, subsets of designed single-stranded fragments are first assembled to give parts of a longer target that may (or may not) have their structures determined. The fragments are subsequently combined to create still longer parts, often by joining sticky ends generated by partial digestion of the ends of the duplex strands [7]. The cycle is then repeated until the full-length L-DNA product is achieved.

With the advent of PCR, this strategy was adapted to the total synthesis of a gene encoding human leukocyte interferon [8] and a gene encoding ribonuclease S protein [9]. The second gene synthesis was the first to exploit the phosphoramidite synthesis approach developed in the Caruther laboratory [3], and also the first to illustrate the ability of total synthesis to generate DNA products that are productively different from what might be found in nature. Total synthesis was used to introduce restriction sites to facilitate subsequent manipulation, add 'watermarks' to track the gene’s provenance, and choose codons to improve the expression of the gene [10].

This combination of automated synthesis of DNA fragments followed by their manual assembly, with PCR used to recover manually assembled partial constructs, began a three decade long effort that has continued to push the limits of synthesis and convergent assembly. One recent example is the synthesis using convergence of a DNA molecule that exactly reproduced (except for watermarks) the natural genome of *Mycobacterium genitalium* [11]. Although the power of synthesis was not exploited to place innovative features into the synthetic *M. genitalium* genome, the use of cells to assist in the assembly of synthetic fragments represented a technological advance.

In all of these efforts, while the synthesis of fragments was automated, the subsequent assembly of parts from them, and the assembly of larger parts from smaller parts, was not. While costs estimates vary, the paper reporting the convergent assembly of the *Mycobacterium* genome had 17 coauthors and represented millions of dollars of expense [11]. More recently, a functional 272,871 base-pair designer chromosome was assembled based on a yeast chromosome [12]. The sequence of chromosome III of *Saccharomyces cerevisiae* was edited with insertions and deletions to give a smaller “synIII” product. This expensive project engaged a team of more than 70 coauthors.

These heroic accomplishments have driven the vision, now called “synthetic biology” [13–17], of converting automatically synthesized short DNA molecules (50–100 nucleotides in length) into much longer DNA constructs by autonomous self-assembly, without the cost of manual convergent assembly. In addition to the DARPA Foundries “1,000 molecules” strategy mentioned above, the United States Army Research Office in 2011 issued a small business grant solicitation seeking software to allow 30,000 base pairs of single-stranded DNA to self-assemble to form nanostructures. In 2012, DARPA issued a small business grant solicitation seeking technology to assemble single-stranded synthetic fragments to give 20,000 base-pair DNA constructs.

If DNA were in fact the idealized molecule taught in introductory biochemistry classes, the specificity of Watson–Crick nucleobase pairing might indeed allow autonomous assembly of large numbers of synthetic single strands to give such large targets, with only a modest amount of design to ensure minimal off-target annealing. With ideal DNA, plasmid-sized constructs (L-DNA, 1,000–10,000 base pairs) and possibly even ultra-long DNA constructs (UL-DNA > 10,000 base pairs) might assemble simply by combining the requisite single-stranded oligonucleotides followed by annealing, primer extension to fill in any gaps, and ligation.

**Figure 1:**
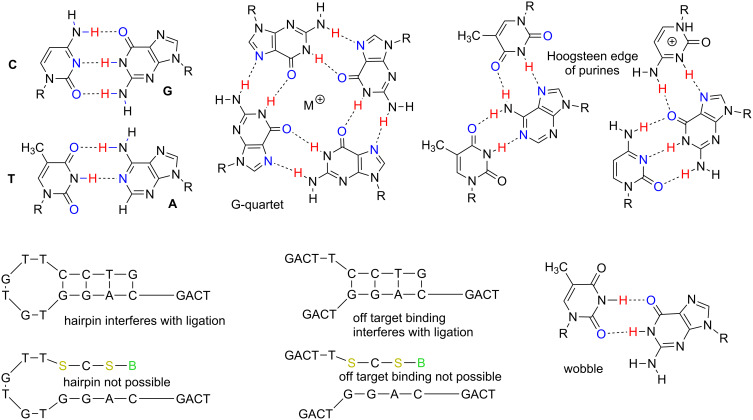
Structures explaining why large DNA (L-DNA) constructs cannot easily autonomously self-assemble from many synthetic single-stranded DNA oligonucleotides. Clockwise from top left, the strong C:G and weak T:A pairs complicate design. G-quartets can arise from G-rich sequences. Major groove interactions involving hydrogen bonding to the “Hoogsteen edge” of purines can compete with Watson–Crickery. Wobble pairs allow formal mismatches to nonetheless contribute to duplex stability, depending on context. The low information density of four-nucleotide DNA allows easy off-target hybridization and unimolecular formation of hairpins. Unimolecular processes (like hairpin formation) compete with the desired intermolecular hybridization especially effectively at low concentrations of oligonucleotide. Adding components of an artificially expanded genetic information system (AEGIS, here **S** and **B**) prevent a class of these obstructive structures.

Unfortunately, DNA is not this ideal. With just four nucleotides, the information density of standard DNA is too low to allow (without explicit design) even a dozen or so single strands to reliably self-assemble upon simple mixing. Even with explicit design, the number of fragments that can be self-assembled appears to be not much larger. This is because of properties intrinsic in the structure of the nucleobases themselves. First, even if rule-based Watson–Crick pairing were the only possible interaction, the presence of “strong” and “weak” G:C and A:T pairs makes design challenging. Also complicating self-assembly of single-stranded DNA fragments are folded single-strand structures (such as hairpins) that compete with desired inter-strand hybridization. A rich repertoire of non-Watson–Crick interactions (e.g., wobble, major groove binding) can also compete with Watson–Crickery (Figure 1).

The work reported here began with the observation that several of the structural features of DNA that intrinsically limit autonomous assembly of standard DNA fragments might be overcome by adding non-standard nucleotides to the repertoire introduced into DNA strands by automated synthesis. These additional non-standard nucleotides come from components of an artificially expanded genetic information system (AEGIS) [18–19].

**Figure 2:**
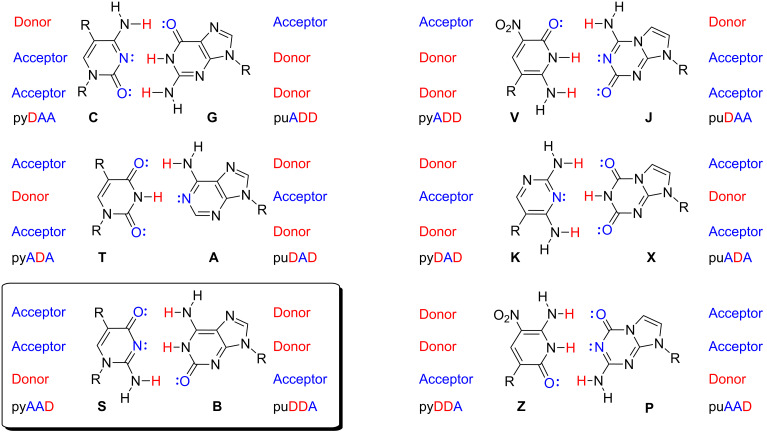
Watson–Crick pairing rules follow two rules of complementarity: (a) size complementarity (large purines pair with small pyrimidines) and (b) hydrogen-bonding complementarity (hydrogen-bond acceptors, A, pair with hydrogen-bond donors, D). Rearranging donor and acceptor groups on the nucleobases, while not changing the geometry of the Watson–Crick pair, creates an artificially expanded genetic information system (AEGIS). AEGIS components add information density to DNA strands built from them. The **S**:**B** pair used in this work is highlighted in the box.

AEGIS adds nucleotides to the DNA alphabet by rearranging the hydrogen-bonding units displayed by the nucleobases, allowing them to pair orthogonally within the geometry of the Watson–Crick pair (Figure 2). With higher information density in AEGIS, more fragments should self-assemble with fewer off-target hybridization, fewer hairpins, and fewer close mismatches that slow down annealing.

Of course, the resulting assembly has unnatural AEGIS components, which may not be desired in the final L-DNA construct. The recently developed conversion technology [20] solves this problem for certain AEGIS pairs. Conversion occurs when polymerases are forced to mismatch a standard nucleotide opposite an AEGIS nucleotide by (a) not being provided the complementary AEGIS triphosphate and (b) exploiting a chemical feature of the AEGIS nucleotide that directs a specific mismatch.

**Figure 3:**
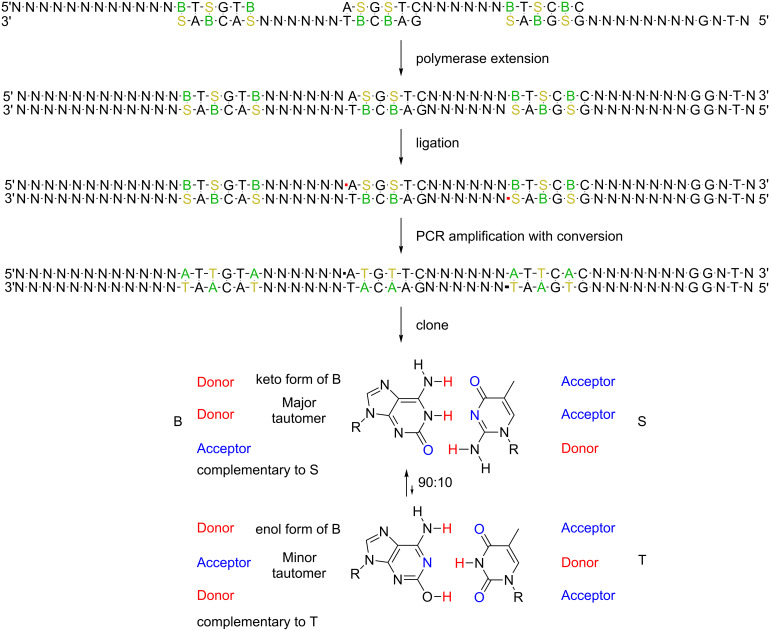
(top) Schematic showing the mix-anneal-extend-ligate-amplify process exploiting AEGIS pairing (to obtain clean assembly) followed by conversion to remove AEGIS pairs from the final construct. The process starts by mixing single-stranded DNA fragments designed to have their ends anneal as duplexes that include **S**:**B** pairs (note the colors). The higher information density enabled by the additional **S**:**B** pair lowers off-target hybridization, eliminates hairpins, and better guides the formation of the desired duplexes. After these duplexes are formed, the 3’-ends are extended using a polymerase that does not displace strands to give nicked DNA. Ligase then seals the nicks by forming the red bonds. Then, PCR amplification with conversion (shown here) or direct transformation into SEGUE^TM^ replaces the **S**:**B** pairs in the construct by T:A pairs. (bottom) **B** in its major tautomeric form pairs with **S**; in its minor tautomeric form, **B** pairs with standard T. This allows the AEGIS **S**:**B** pair to support the assembly of multiple single-stranded fragments using the mix-anneal-extend-ligate-amplify process, to be followed by conversion of the **S**:**B** pairs to T:A pairs after two cycles of PCR. In the conversion template **B** is replaced by standard A via an intermediate **B**:T mispairing, while template **S** is replaced by T via an intermediate **S**:**B** pairing followed by a second intermediate **B**:T mispairing.

For example, when the AEGIS nucleotide 2’-deoxy-5-methylisocytidine (trivially designated **S**) and its AEGIS complement 2’-deoxyisoguanosine (trivially designated **B**) (Figure 2) are used to assemble a L-DNA construct, the **S**:**B** pair forms orthogonally to the T:A and C:G pairs [18–19]. However, if d**S**TP is missing during primer extension, **B** in a template must be mismatched during primer extension. Here, conversion exploits the ability of a minor tautomer of **B** to mismatch with standard T (Figure 3), After this initial mismatch, the misincorporated T directs the incorporation of dATP, resulting in a net conversion of the **S**:**B** pairs in the preliminary construct to T:A pairs in the final construct (Figure 3) [20]. This conversion can be performed in vitro, but also in *E. coli* in a strain (SEGUE) being developed to replicate plasmids containing expanded genetic systems.

**Figure 4:**
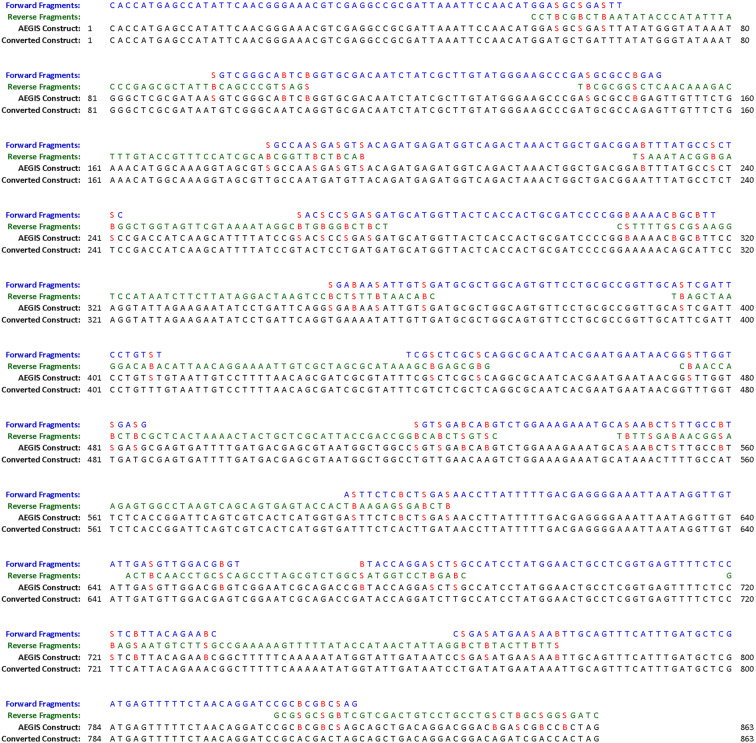
The exact sequences and overlaps of the fragments designed by OligArch to allow the autonomous assembly of a gene encoding an aminoglycoside 3’-phosphotransferase that confers upon *E. coli* resistance to kanamycin, aligned above the gene that arises via conversion PCR. The AEGIS nucleotides, **S** and **B** (red), were placed in the overlap regions to guide self-assembly.

Here, we illustrate this strategy by performing a total synthesis of a gene encoding an aminoglycoside 3’-phosphotransferase that confers kanamycin resistance by autonomous self-assembly, where **S**:**B** pairs used to create the clean fragment assembly are later converted via this process to T:A pairs. Figure 4 shows the details of the assembly.

## Results

### The OligArch software package and the construct design

To support this project, a software package (OligArch) [21] was created to design DNA molecules that exploit AEGIS nucleotides to facilitate the self-assembly of multiple single-stranded DNA. The OligArch package takes as input a target sequence for a desired long DNA (L-DNA) construct. It then fragments the target to deliver, as output, a set of single-stranded DNA molecules that include AEGIS components. OligArch designs these fragments so that, after they are annealed, the annealed fragments are extended by a DNA polymerase to fill in any gaps, the nicks in the resulting duplex are ligated (Figure 3 schematically, Figure 4 in detail), and the AEGIS pairs are replaced by standard pairs by conversion PCR, all to produce the desired L-DNA construct to emerge with only standard bases. To meet these performance specifications, OligArch must know the rules for conversion of AEGIS nucleotides to standard nucleotides; these are provided from experimental data.

OligArch also allows the user to designate certain regions as protein-encoding sites; at these sites, redundancy in the genetic code can be exploited. Sites may also be designated where no flexibility is permitted. OligArch seeks to have desired duplexes formed with a melting temperature not lower than a user-specified threshold temperature. Once this temperature is specified, OligArch automatically designs the fragments so that the best off-target hybridizations have melting temperatures below a second “off-target” level.

This difference (or “spread”) between the melting temperature of the “weakest wanted” and “strongest unwanted” pairings is a predictor of the success of autonomous assembly; the larger the spread, the more likely the self-assembly will succeed. With just the four standard nucleobases, the spread cannot remain large as the number of fragments increases. Accordingly, various practitioners recommend attempting self-assembly with no more than a dozen or so fragments [22], although circa three dozen have been assembled inside of yeast cells [23].

In this work, we chose **S** (2’-deoxy-5-methylisocytidine) and **B** (2’-deoxyisoguanosine) as the AEGIS nucleotides; adding these gives a six-letter GACT**SB** DNA alphabet. This was an alternative to the AEGIS nucleotides 2-amino-8-(1’-β-D-2’-deoxyribofuranosyl)imidazo[1,2-*a*]-1,3,5-triazin-4(8*H*)-one (trivially named **P**) and 6-amino-5-nitro-3-(1’-β-D-2’-deoxyribofuranosyl)-2(1*H*)-pyridone (trivially named **Z**) (Figure 2), which give a GACT**ZP** six-letter DNA alphabet. This choice reflected simpler conversion rules, to be discussed elsewhere.

### AEGIS-guided oligonucleotide assembly yields a kanamycin resistance gene

The actual sequences designed by OligArch and used here are shown in Figure 4, with overlaps. These were prepared by automated DNA synthesis from six phosphoramidites (four standard and two AEGIS). They were then mixed in equal amounts, heated and cooled. The 3’-fragments were then extended at 48 °C using Phusion DNA polymerase to give a nicked construct and the nicks were sealed with ligase.

**Figure 5:**
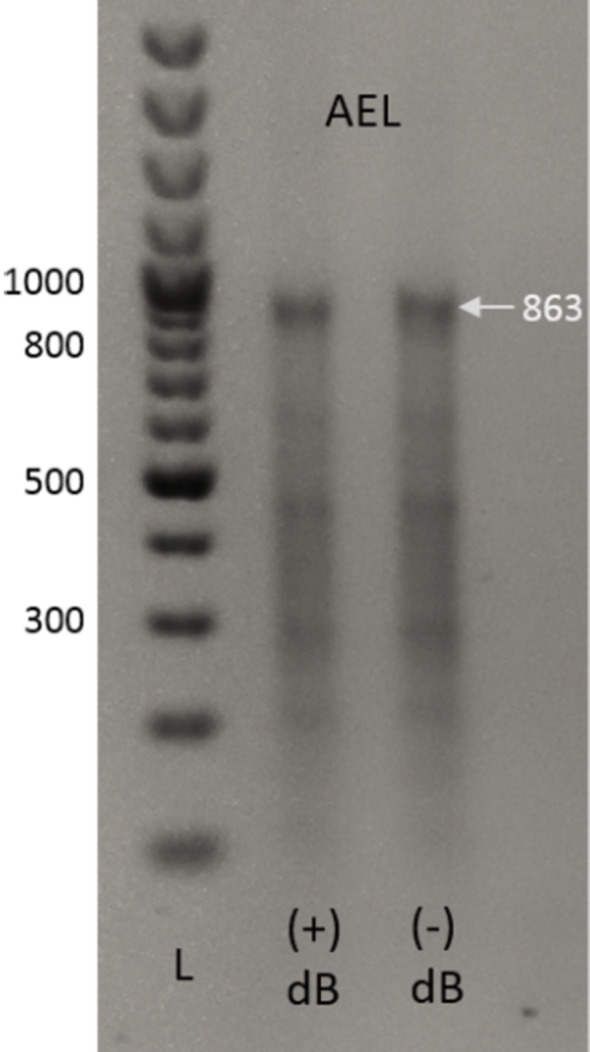
Agarose gel (1%, stained with ethidium bromide) showing the products (before PCR) arising from the autonomous assembly of the AEGIS-containing oligonucleotide fragments (Figure 4) for a gene encoding kanamycin resistance (863 base pairs) under two incubation conditions (#1, same method as in text with 2’-d-isoGTP (d**B**TP) included in the mixture; #2 as in text). Left lane (L) is a ladder with markers indicated at 300, 500, 800, and 1,000 nucleotides. Remarkably, a principal product runs at 863 nucleotides, the length of the complete construct arising from the autonomous assembly of 20 fragments. When similar autonomous assembly without AEGIS nucleotides is attempted, the desired full-length product is almost never seen as a principal product, and almost never recovered without PCR.

The first indication that the GACT**SB** AEGIS self-assembly was successful was the electrophoretic detection of the full-length non-amplified product (Figure 5). A gel resolving crude, non-amplified products obtained from the “one-pot” annealing, extension, and ligation process showed a major band at ~863 base pairs, the size of the expected product (Figure 5). This suggested that the 20 fragments with AEGIS overhangs self-assembled to give the target gene as one of the principal products. As discussed in our comparative study (see below), this is rarely seen in mixtures of fragments built from the standard GACT DNA alphabet, even with fewer fragments.

**Figure 6:**
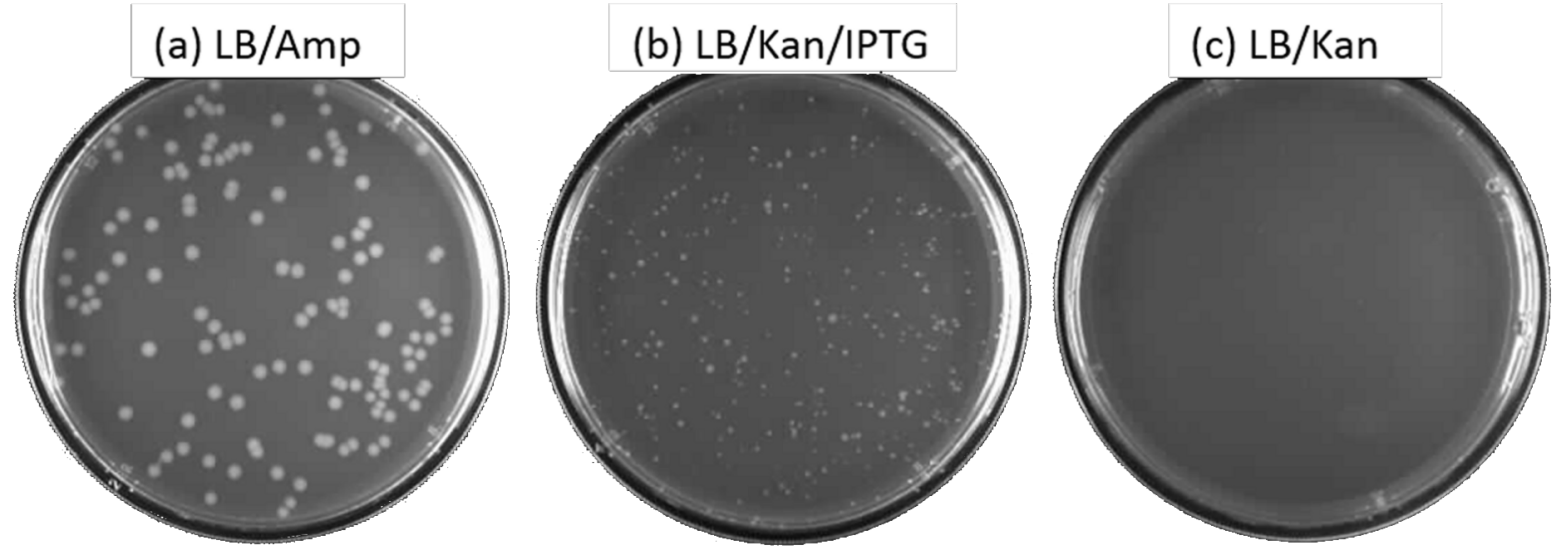
LB-agar plates spread, at various dilutions, with 25 µL cells transformed by plasmids containing the self-assembled kanamycin-resistance gene placed behind a β-galactosidase promoter in the TOPO expression vector (Invitrogen), but without selection for resistance to kanamycin. (a) The cell culture was diluted in two steps by a factor of 267,000. Then, aliquots (25 µL) were plated on agar containing only ampicillin. Replica plates (only one is shown) gave 123 and 99 colonies (average is 111 colonies), for a calculated 29.0 × 10^6^ ampicillin-resistant colonies/mL in the original cell culture. (b) The cell culture was diluted by a factor of 2. Then, 25 µL was plated on agar containing IPTG and kanamycin. Replica plates (only one is shown) gave 308 and 276 colonies (average is 292 colonies), for a calculated 23,000 kanamycin-resistant colonies/mL in the original cell culture. (c) With insert but without IPTG induction of the gene for kanamycin resistance, no growth is seen.

The product of the autonomous assembly was then ligated behind a β-galactosidase promoter in a plasmid containing a gene conferring resistance to ampicillin. This was used to transform *E. coli* cells, which were found to grow in medium containing kanamycin. Plating experiments quantitated these results (Figure 6). Plates with kanamycin but no IPTG (which induces expression of the synthetic gene) gave no colonies (Figure 6c). With IPTG, however, multiple colonies grew (Figure 6b) in the presence of kanamycin, with the expected smaller size than the colonies seen on plates lacking kanamycin altogether (Figure 6a).

These results demonstrated successful autonomous assembly of a gene encoding an active aminoglycoside 3’-phosphotransferase. They allowed us to estimate the success of the overall “start-to-finish” process, including insertion into the plasmid and other steps unrelated to the OligArch GACT**SB** AEGIS assembly. The primary culture contained approximately 23,000 kanamycin-resistant cells per mL. The same culture contained approximately 29,000,000 total cells per mL. This suggests that the overall process generated successfully constructed, inserted, converted, and successfully expressed kanamycin-resistance genes in ~0.1% of the cells that had acquired ampicillin resistance by transformation.

The final demonstration of the success of the GACT**SB** autonomous assembly came, of course, from the sequencing of the cloned product. Sequencing also allowed us to estimate the error associated with the process. Table S1 (Supporting Information File 1) collects a set of sequences of the constructs that were created by the procedure where d**B**TP was used in the conversion procedure (see Experimental). While 1–2% errors were seen, none were found in the sites where **S**:**B** pairs had been present. The errors were found most frequently at the ends of the reads, as expected for sequencing errors, rather than errors in the primary synthesis, polymerase extension, or conversion PCR.

### Standard oligonucleotide assembly attempts without AEGIS components fail

While these results show that synthetic DNA fragments can self-assemble to give complete genes, they also raise the question: Are AEGIS components needed? Can we not assemble genes of the same size with only standard nucleotides, without any AEGIS components at all?

It appears that no systematic experiments have been performed to benchmark the limits to autonomous self-assembly of natural DNA. Therefore, we undertook a series of “push to fail” experiments where we attempted to prepare larger and larger assemblies from more and more fragments constructed from standard nucleotides, using three sets of fragments with up to 32 fragments in each set. Each set of fragments was designed by OligArch with only one goal in mind: to give autonomous assembly the best chance of success. To this end, OligArch used three different “seeds” to initiate the fragment design [21]. The fragments were designed to have nearly identical lengths (50–52 nts) and 15–17 nucleotide overlap with melting temperatures predicted to lie in a narrow range (44–56 °C; calculated without magnesium). OligArch also designed the sequences to have no-off target hybrids having a melting temperature greater than 25 °C, a full 20 °C below that predicted for the desired annealing pairs.

Two of the three constructs (“32B” and “32C”) contained the four standard nucleotides, G, A, T, and C throughout. In the third construct (“32A”), OligArch placed AEGIS nucleotides **S** and **B** (Figure 2) in the overlapping regions to facilitate self-assembly. Figures S1–S3 (Supporting Information File 1) show the designed single-stranded DNA fragments and their hybridizing segments. All three constructs were designed to have approximately 1.1 kb pairs when fully assembled (Supporting Information File 1, Tables S7–S9).

**Figure 7:**
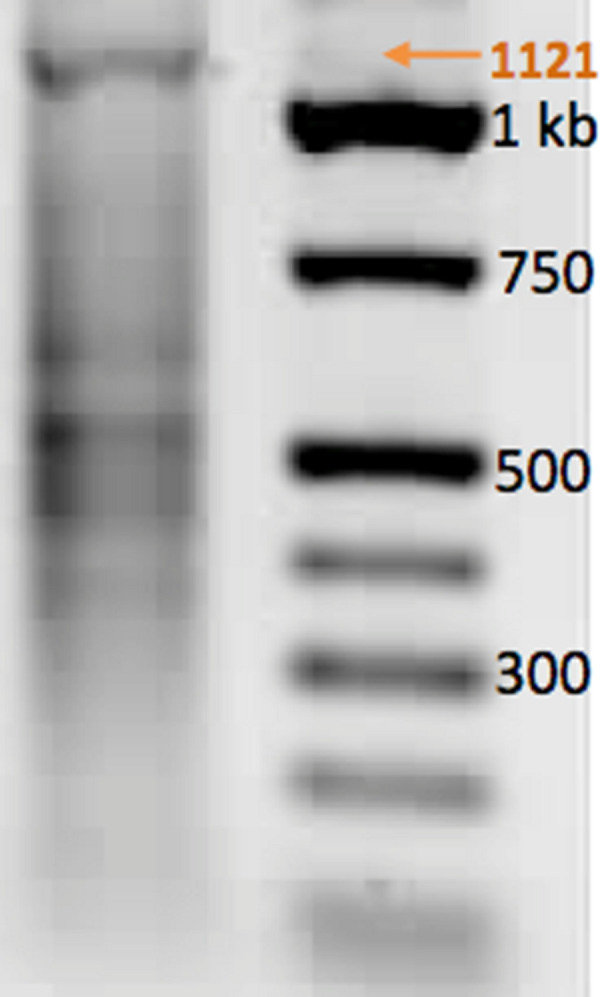
(left) Autonomous assembly of the 32A construct yielded the desired 1,121 base-pair amplicon in a single step involving mixing, annealing, extending, and ligation, followed by PCR amplification (30 cycles). Orange arrow shows the full length product obtained. (right) Ladder of synthetic fragments having lengths indicated (nucleotides).

The results are consistent with the discussion above. With the 32A construction (Supporting Information, Figure S1) that did exploit the AEGIS **S**:**B** pair (Figure 2) in the annealing overhang segments, a PCR product arising from end-to-end PCR (with conversion of the **S**:**B** pairs to T:A pairs) with the expected length of 1,121 base pairs was immediately seen (Figure 7). The product was directly cloned and sequenced, to prove success of the AEGIS-assisted autonomous assembly.

**Figure 8:**
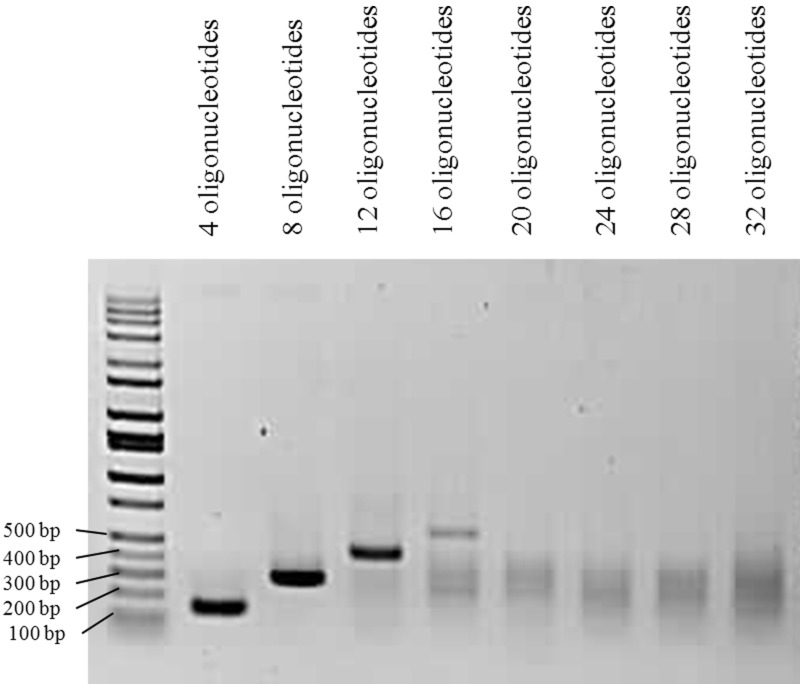
Fragments built only from standard nucleotides, without AEGIS, and designed by OligArch [21] to support the 32B assembly (Supporting Information File 1, Figure S2) resulted in successful self-assembly of up to 16 pieces, in decreasing amounts as the constructs became longer, where the 16-fragment assembly was recovered by PCR. However, under these conditions PCR failed to recover longer assemblies built from 20, 24, 28, or 32 fragments. This represents an experience common when attempting autonomous assembly of DNA single strands. Shown is a 1% agarose gel resolving those products, with ladder at left.

With the 32B construct that lacked AEGIS nucleotides, no full-length product was observed when all 32 fragments were mixed without amplification (data not shown). To rule out the possibility that the oligonucleotides were defective, smaller constructs were self-assembled. Figure 8 shows the results of stepwise assembly of subsets of the fragments, after the target ligation products were rescued from the mixture by PCR (30 cycles). As is evident by the intensity of the bands arising from the amplicons four, eight, and twelve fragments easily assembled without the need for any AEGIS nucleotides to guide that assembly. Sixteen fragments also assembled, but the yield of the assembled construct was approximately 30% lower. An assembly of 20 fragments could be only be recovered by PCR under different conditions (data not shown). Attempts to assemble 24 to 32 fragments failed to yield any detectable amplicon.

However, the two halves of the 1,135 base-pair construct, once separately assembled and amplified by PCR to create the half assemblies in large amounts, could then be manually joined in a standard convergent synthesis after blunt-end ligation. The desired 1,135 base-pair target construct was recovered by PCR (see Supporting Information File 1, Figure S4). This process was, of course, not automated; it represented the same stepwise convergent assembly of L-DNA that has been reported previously [3–5,9].

The 32C assembly attempt also failed initially. However, upon increasing the concentrations of oligonucleotide fragments from 62.5 nM to 125 nM, followed by PCR amplification, a PCR product of the desired length could occasionally be recovered (Supporting Information File 1, Figure S5).

## Discussion

Adding nucleotide “letters” to an expanded genetic “alphabet” increases the information density of the resulting DNA sequences. With four nucleotides, the number of overlapping 15mers is ca. 1.1 billion (≈4^15^). While this number might appear to be large, it includes an enormous range of melting temperatures, because the G:C and A:T pairs contribute differently to the stability of a duplex. Adding two additional nucleotides increases the number of potential overlaps to 470 billion (≈6^15^), more than 400 fold higher.

The increased information density is expected *a priori* to improve the ability of single-stranded fragments to self-assemble without human guidance. Further, the **S**:**B** pair with its three hydrogen bonds is “strong,” like the G:C pair. This means that the OligArch design of fragments can focus on obtaining the best chance of delivering the longest possible desired sequence, rather than accommodating another weak nucleobase pair.

Further, **S** and **B** can be used strategically, as illustrated in the synthesis of the kanamycin gene. Here, **S** and **B** were placed at the ends of the single-stranded oligonucleotides, and not in the central portions of those oligonucleotides. This makes it essentially impossible for intramolecular hairpins to compete with the intermolecular annealing (Figure 1). Intramolecular folding is expected to be especially destructive as the number of fragments increases, if the concentration of each individual fragment decreases.

In “push to fail” experiments that compared the assembly of fragments that exploit AEGIS components with assemblies that do not, the improvement could be assessed. Thus, the 32B assembly of entirely standard oligonucleotides, designed with no constraint other than to optimize self-assembly, worked with up to 16 fragments (but not longer) (Figure 8). With higher concentrations of fragments (125 nM), the 32C assembly, upon PCR rescue, sometimes yielded constructs more easily than the 32B assembly (Supporting Information File 1, Figure S5). While folds and other non-canonical secondary structures (Figure 1 and Supporting Information File 1, Figure S6) may have prevented the 32B assembly from working, comparable folds can be found for any set of fully standard fragments.

Further, real sequences intended to advance a synthetic biology agenda are rarely ideal; their design is likely constrained in a way that, without the help of AEGIS nucleotides, will prevent OligArch from achieving the large differences in melting temperatures, and achieving total avoidance of hairpins and other secondary structures that obstruct autonomous assembly.

These results provide, therefore, a realistic view of what automated DNA synthesis can provide by way of products that can be carried forward in an automated fashion to make larger products. The use of AEGIS nucleotides enables a breakthrough of the “dozen fragment barrier” and facilitates the modular formation of L-DNA constructs (>1 kb). Other factors will now become limiting for the self-assembly of L-DNA constructs, including errors in the synthesis of the DNA fragments used for the assembly. These must be mitigated in any effort to look forward, especially in the hope of finding inexpensive automated strategies that assemble inexpensive oligonucleotides into long DNA constructs (L-DNA) and (possibly) ultralong DNA (UL-DNA) constructs, including whole chromosomes.

## Experimental

### Materials and methods

#### Design

The sequence for an aminoglycoside 3’-phosphotransferase that encodes resistance to kanamycin is commonly used in cloning vectors and was obtained from the National Center for Biotechnology Information database (NCBI). Using the OligArch software designed for this purpose [21], a gene that encodes the protein was constructed to be assembled from 20 single-stranded DNA fragments containing two, three, or four **S** (2’-deoxy-5-methylisocytidine) and **B** (2’-deoxyisoguanosine) nucleotides at their 5’ and 3’ ends. To obtain consistent melting temperatures, OligArch designed oligonucleotides ranging in size from 41 to 68 nucleotides (nts) (Table 1). Overlaps were 11–15 nucleotides in length, and designed to have theoretical melting temperatures of 42 and 52 °C (calculated at 50 mM NaCl without divalent magnesium; using the nearest neighbor formula from http://www.clinchem.org/content/45/12/2094.long; melting temperatures in 10 mM MgCl_2_ are expected to be ~10 °C higher) The **S**:**B** pair was assumed to contribute to duplex stability the same as the C:G pair. Enthalpy and entropy values for nucleotide pairs in context were taken from [24].

**Table 1:** Oligonucleotides containing **S** (2’-deoxy-5-methylisocytidine) and **B** (2’-deoxyisoguanosine) used in this work.

Name	Sequence (5’–3’)

01KanR 1_66	CACCATGAGCCATATTCAACGGGAAACGTCGAGGCCGCGATTAAATTCCAACATGGASGCSGASTT
02KanR 55_106	SGASTGCCCGACBTTATCGCGAGCCCATTTATACCCATATAABTCBGCBTCC
03KanR 94_151	SGTCGGGCABTCBGGTGCGACAATCTATCGCTTGTATGGGAAGCCCGASGCGCCBGAG
04KanR 141_193	BACBTCBTTGGCBACGCTACCTTTGCCATGTTTCAGAAACAACTCSGGCGCBT
05KanR 181_242	SGCCAASGASGTSACAGATGAGATGGTCAGACTAAACTGGCTGACGGABTTTATGCCSCTSC
06KanR 228_276	TCBTCBGGBGTBCGGATAAAATGCTTGATGGTCGGBAGBGGCATAAAST
07KanR 265_318	SACSCCSGASGATGCATGGTTACTCACCACTGCGATCCCCGGBAAAACBGCBTT
08KanR 306_362	CBACAATBTTSTCBCCTGAATCAGGATATTCTTCTAATACCTGGAASGCSGTTTTSC
09KanR 349_407	SGABAASATTGTSGATGCGCTGGCAGTGTTCCTGCGCCGGTTGCASTCGATTCCTGTST
10KanR 393_449	GBGCGAGBCGAAATACGCGATCGCTGTTAAAAGGACAATTACABACAGGAATCGABT
11KanR 439_485	TCGSCTCGCSCAGGCGCAATCACGAATGAATAACGGSTTGGTSGASG
12KanR 474_530	CSTGSTCBACBGGCCAGCCATTACGCTCGTCATCAAAATCACTCGCBTCBACCAABC
13KanR 520_560	SGTSGABCABGTCTGGAAAGAAATGCASAABCTSTTGCCBT
14KanR 546_604	BTCBAGSGAGAABTCACCATGAGTGACGACTGAATCCGGTGAGAASGGCAABAGSTTBT
15KanR 592_657	ASTTCTCBCTSGASAACCTTATTTTTGACGAGGGGAAATTAATAGGTTGTATTGASGTTGGACGBGT
16KanR 643_686	CBAGBTCCTGGTASCGGTCTGCGATTCCGACSCGTCCAACBTCA
17KanR 673_734	BTACCAGGASCTSGCCATCCTATGGAACTGCCTCGGTGAGTTTTCTCCSTCBTTACAGAABC
18KanR 720_778	STTBTTCATBTCBGGATTATCAATACCATATTTTTGAAAAAGCCGSTTCTGTAASGABG
19KanR 765_832	CSGASATGAASAABTTGCAGTTTCATTTGATGCTCGATGAGTTTTTCTAACAGGATCCGCBCGBCSAG
20KanR 822_863	CTAGSGGSCGBTCSGTCCGTCCTGTCAGCTGCTBGSCGSGCG

Additionally, the L-DNA product was designed to have a CACC tetranucleotide immediately upstream from the start codon; this assisted incorporation into a TOPO expression vector. The synthetic segment was also designed to have a *Bam*HI region downstream of the stop codon, also to assist in downstream processing. Thus, the complete target L-DNA sequence had 863 base pairs.

#### Synthesis

The oligonucleotides containing **S** and **B** shown in Table 1 were obtained from IDT, where they were prepared by automated solid phase phosphoramidite-based synthesis following the procedure of Jurczyk et al. [25]. Each oligonucleotide was purified by polyacrylamide gel electrophoresis (PAGE).

#### Annealing, extending and ligating the preselected DNA oligonucleotide fragments

The lyophilized synthetic fragments were resuspended in buffer (10 mM Tris-HCl, pH 8.0, 0.1 mM EDTA) to give stock solutions, each holding 10 µM of DNA (by UV spectroscopy at 260 nm prior to dilution). For the annealing step, aliquots (1 µL, containing each 10 pmol, ca. 125–250 ng of DNA) were combined from each of the stock solutions with 5× ISO buffer (8 μL, 25% PEG-6000, 500 mM Tris-HCl, pH 7.5, 50 mM MgCl_2_, 50 mM DTT, 5 mM NAD^+^) and diluted with water (40 μL final volume; final concentrations: 250 nM each oligonucleotide, 5% PEG-6000, 100 mM Tris-HCl, pH 7.5, 10 mM MgCl_2_, 10 mM DTT, 1 mM NAD^+^).

This mixture was then heated at 95 °C for 5 min, and then slowly cooled (0.1 °C/second) to 42 °C. Aliquots (5 μL) of the annealed mixture were transferred to a new tube, and then diluted with a mixture of enzymes (total volume of mixture was 15 µL) containing Phusion^TM^ DNA polymerase (1 U, New England Biolabs, final 0.07 U/µL), *Taq* DNA ligase (400 U, New England Biolabs, final 27 U/µL), ISO buffer, and dNTPs (final 0.2 mM). The mixtures were then incubated at 48 °C for 60 min, cooled and stored at 4 °C.

The annealed-extended-ligated products were resolved on a 1% agarose gel (Figure 5). A major product appeared with a size of ~863 base pairs, the length of the fully assembled target L-DNA product.

#### PCR amplification with conversion of the AEGIS-containing L-DNA product

“Conversion PCR” [25–26] of the assembled product was then carried out using *Taq* Full DNA polymerase (Clontech), which lacks proofreading ability. This leads to the replacement of **S**:**B** pairs by T:A pairs, presumably via the “minor enol tautomer” mechanism shown in Figure 3.

Two conditions were tested for the conversion PCR. The first included a small amount of 2’-d-isoGTP (d**B**TP, ChemGenes, 0.3 µL of a 10 mM stock, 3 nmol per assay, final concentration 60 µM) to complement the 2’-d-isoC in the template. In the presence of d**B**TP, the conversion requires two cycles. In the first, d**B**TP is incorporated opposite template d**S**, while dTTP is mismatched opposite template d**B**, presumably via its minor tautomer. In the second cycle, conversion is completed by mismatching dT opposite the minor enol d**B** in the newly synthesized template (Figure 3). Thus, the use of d**B**TP allows the PCR to proceed without requiring the polymerase to mismatch a standard nucleotide opposite template d**S**, a mismatch that is not assisted by any minor tautomer.

A second incubation omitted d**B**TP. This required that in the first cycle, the polymerase must mismatch both dT opposite d**B** and dA opposite d**S**. As shown, these conditions produced lower yields of product, and are not recommended. Nevertheless, this result is consistent with our model for the conversion of **S**:B pairs to T:A pairs.

The annealed-extended-ligated (AEL) sample was used directly in the conversion PCR, without clean-up or dilution. Aliquots of the AEL sample (2 µL) were added to the pre-mix (48 µL) of reagents and enzymes (Supporting Information File 1, Table S2) optionally containing d**B**TP in PCR tubes (200 µL). Water was added for the negative controls. The PCR cycling program was: 95 °C for 2 min, followed by 30 cycles of 95 °C for 40 s, 55 °C for 20 s, and 72 °C for 2 min, with a final extension at 72 °C for 10 min.

As the reverse primer was designed to be upstream of the terminal AEGIS bases, the final PCR product was 14 bp shorter than the initial construct. The bands corresponding to the 849 bp amplicons were excised and purified with the Wizard SV Gel Clean-up System (Promega). Concentrations of amplicon DNA were estimated by their absorbance at 260 nm.

#### Secondary PCR

The L-DNA construct was then prepared for insertion into plasmid vector. Because *Taq* DNA polymerase adds untemplated 3’-adenosines that interfere with incorporation of insert into a directional expression vector, a second PCR was carried out with a non-*Taq* polymerase (PrimeSTAR^TM^, Clontech) using the gel-purified amplicon from the first PCR as its template. Three samples were prepared for secondary PCR: the product prepared by conversion PCR with d**B**TP, the product prepared by conversion PCR without d**B**TP, and a negative control lacking template. PCR reagents were pre-mixed (Supporting Information File 1, Table S3). Aliquots (49 µL) were delivered to 0.2 mL PCR tubes, template or water (1 µL) was added to give a final volume of 50 µL, and PCR was initiated following the program recommended by the supplier of the PrimeSTAR polymerase: hot start at 95 °C for five min followed by 30 cycles of: 98 °C for 10 s, then 68 °C for 20 s. The amplicon was recovered using the QIAquick PCR Purification kit (Qiagen).

#### Incorporation into the vector

Each cleaned-up amplicon (+d**B** and −d**B**, 4 µL) was mixed with TOPO pET101 directional expression vector (1 µL, Invitrogen) and salt solution (1 µL, provided with vector) according to the manufacturer’s recommendation, incubated at room temperature for 10 min, then transferred to wet ice until used to transform competent cells. The insert/vector mixtures were used to transform BL21 Star (DE3, Invitrogen) cells using heat-shock following the manufacturer’s instructions. For each sample type, a fraction of the DNA/vector (3 µL) was added to cells (50 µL, provided with vector kit), and the mixture was incubated on ice for 30 min. The cells were then shocked by heating (42 °C, 35 s) in a water bath. Media (SOC from Invitrogen, 250 µL, provided with vector kit) was then added, and the mixture was placed on ice for 5 min. The mixtures were then transferred to Falcon tubes (15 mL) and incubated (37 °C, shaking) for 1 h. The entire volumes were then transferred by pipetting to pre-warmed (37 °C) LB medium containing ampicillin (100 μg/mL, without kanamycin) and incubated overnight (37 °C). These conditions allowed cells containing the plasmid to grow, whether or not the plasmid contained a functioning inserted gene encoding kanamycin resistance (or, for that matter, any insert at all). To assess whether the assembly had produced a gene able to confer kanamycin resistance, these cells were subcultured in shaken growth medium containing kanamycin and isopropyl β-D-1-thiogalactopyranoside (IPTG). Continued growth was observed, and viability was measured by following an increase in optical density (OD) as a function of time. Negative controls (cells that lacked the kanamycin resistance gene) did not survive overnight incubation in the presence of kanamycin.

Additionally, each insert/vector mixture (+d**B** and −d**B**, 2 µL each) was used to transform TOP10 competent cells (25 µL each, provided with vector kit), a more stable cell line for culturing and maintenance. The cells/DNA mixture was incubated on wet ice for 8 min, shocked at 42 °C for 35 s, and returned to the ice briefly. Room temperature SOC media (250 µL) was immediately added and the cells transferred to a culture tube and incubated at 37 °C with shaking for 1 h. After incubation the TOP10 cells were divided between two LB/Ampicillin (100 µg/mL) agar plates (100 and 175 µL) and incubated at 37 °C overnight. After incubation the plates were stored at 4 °C until prepared for sequencing reactions.

#### Quantitative estimates of the level of success of the OligArch L-DNA construction

Cells from the growth in shaken culture in the presence of kanamycin were used to inoculate a new culture (3 mL) of LB medium containing ampicillin (100 µg/mL) and incubated with shaking at 37 °C until OD reached 1.4. Volumes (25 and 50 µL) of this culture were then plated (with appropriate serial dilutions) on agar containing ampicillin alone (to count cells that had been transformed regardless of the success of the OligArch assembly), kanamycin and isopropyl β-D-thiogalactoside (IPTG, 1 mM, which induced the expression of any kanamycin resistance gene carried by the plasmid), and kanamycin without IPTG (the negative control). Without IPTG, any kanamycin resistance genes are not induced, and no cells were expected to survive kanamycin.

#### Sequencing the L-DNA construct that confers kanamycin resistance

To prepare plasmids for sequencing of cells with selective pressure to contain the insert that correctly encodes for kanamycin resistance, cells previously found to be viable in shaken culture in the presence of kanamycin were plated on LB/agar plates containing kanamycin (100 µg/mL) and IPTG (1 mM). Single colonies were then used to inoculate cultures grown in the presence of ampicillin alone. Plasmids were harvested with the Zyppy™ Miniprep kit (Zymo Research) and were sent out to be sequenced with vector primers (T7 Terminator and T7 Promoter long). The sequences obtained were then analyzed (Supporting Information File 1, Table S4).

To prepare plasmids with no selective pressure, which would potentially include inserts that contained assembly errors or base substitutions arising during PCR conversion other than **S**:T and **B**:C, colonies from the TOP10 culture plates were used to inoculate cultures grown in the presence of ampicillin alone. Plasmids were harvested as described above and sequenced with vector primers (BioBasic).

## Supporting Information

File 1 Additional Information.
