# Supplementary material for: Autonomous assembly of synthetic oligonucleotides built from an expanded DNA alphabet. Total synthesis of a gene encoding kanamycin resistance
Source: Beilstein J Org Chem. 2014 Oct 9;10:2348–60. doi: 10.3762/bjoc.10.245 (PMC4222377; doi:10.3762/bjoc.10.245)
Supplement: File 1 — Additional Information. [file Beilstein_J_Org_Chem-10-2348-s001.pdf]

Supporting Information

for

Autonomous assembly of synthetic oligonucleotides built from an expanded DNA alphabet. Total synthesis of a gene encoding kanamycin resistance

Kristen K. Merritt[1,2], Kevin M. Bradley[1,2], Daniel Hutter[1,2,3], Mariko F. Matsuura[1,2,4], Diane J. Rowold[1,2], and Steven A. Benner[1,2,3]*

Address: [1]Foundation for Applied Molecular Evolution, P.O. Box 13174, Gainesville, FL, 32604, [2]The Westheimer Institute for Science and Technology, 720 S. W. 2nd Avenue, Suites 201-208, Gainesville, FL, 32601, [3]Firebird Biomolecular Sciences LLC, 13709 Progress Blvd. Box 17, Alachua, FL 32615 and [4]Department of Chemistry, University of Florida, Gainesville, FL, 32611

Email: Steven A. Benner - sbenner@firebirdbio.com

* Corresponding author

Additional Information

Fragment design for three "push to fail" constructs

In independent experiments (and in two different physical locations), three of the coauthors attempted the autonomous assembly of three long-DNA (L-DNA) constructs from synthetic DNA fragments designed by the OligArch software tool [2]. All three constructs were designed to have approximately 1100 nucleobase pairs and arise via self- assembly of 32 single stranded DNA fragments (see **Figures S1**, **S2** and **S3** and **Tables S7**, **S8**, and **S9**). The target constructs had no function at all which allowed their designs to have, as their only goal, successful autonomous self-assembly. OligArch generated these three sets of sequences by using three different "seeds" to initiate the fragment design.

The 32 fragments were designed by the OligArch software to have nearly identical lengths (50-52 nts) with 15-17 nucleotide overlaps having melting temperatures predicted to lie in a narrow range (44 - 56 °C). The sequences were programmed to form no-off target hybrids having a melting temperature greater than 25 °C, a full 20 °C below that predicted for the desired annealing pairs. Two of the three constructs ("32B" and "32C") contained only the four standard nucleotides, G, A, T, and C. In the third construct ("32A"), OligArch placed AEGIS nucleotides S and B (**Figure 2** of the principal manuscript) in the overlapping regions to facilitate self-assembly. **Figures S1**, **S2**, and **S3** show the designed oligonucleotides aligned to show their hybridizing segments. The gaps were subsequently filled in by DNA polymerase to yield nicked constructs, and the nicks were sealed by ligase.

Annealing extension and ligation

The oligonucleotide fragments were prepared by automated DNA synthesis and quantitated by UV spectroscopy. The oligonucleotides used for 32B and 32C constructs were ordered from Integrated DNA Technologies (IDT, Coralville, IA, USA). The oligonucleotides used for 32A

construct were ordered from Firebird Biomolecular Sciences (Gainesville, FL, USA). Self-assemblies of constructs were attempted in stages by annealing, extension and ligation (AEL) of various subsets of the total fragment set as outlined below.

(a) Annealing: An annealing solution (40 µL) was prepared by mixing equal concentrations of each synthetic oligonucleotide (1 µL of 20 µM unless otherwise stated) and 1X ISO reaction buffer (5% PEG-8000, 100 mM Tris-HCl, pH 7.5, 10 mM $MgCl_2$, 10 mM DTT, 1 mM NAD^+). The mixture was then heated to 80 °C for 5 min, and the temperature was then reduced at 0.1 °C/sec to 40°C (32C/32B) or 42 °C for 30 minutes (32A).

(b) Extension and ligation: Unless otherwise stated, the extension and ligation proceeded as follows: An enzyme mixture (15 µL) was created in 1X ISO reaction buffer (5% PEG-8000, 100 mM Tris-HCl, pH 7.5, 10 mM $MgCl_2$, 10 mM DTT, 1 mM NAD^+) with 0.05 U/µL Phusion® High-Fidelity DNA Polymerase, 2.0 U/µL *Taq* DNA Ligase, and 0.2 mM dNTPs. This mixture was added to annealed sample (5 µL). Then samples were incubated at 40 °C for 30 min (32C/32B) or 48 °C for 60 minutes (32A).

Downstream analysis:

(a) PCR amplification: To analyze the success of the assemblies of subsets and full sets of the synthetic fragments, PCR was performed with the appropriate primers (**Tables S10**, **S11** and **S12**) in reaction mixtures (50 µL) containing 1X *Taq* buffer (10 mM Tris-HCl (pH 8.3), 50 mM KCl, 1.5 mM $MgCl_2$), 0.2 mM dNTPs, 0.4 µM forward/reverse primer sets, and 0.04 U/µL *Taq* polymerase) and 1 µL of the (putatively) ligated oligonucleotides. The following cycling conditions were used: for the 32C/32B constructs, 95 °C for 1 minute, followed by 30 cycles of 95 °C for 20 seconds, 50 °C for 20 seconds, and 72 °C for 90 seconds; for the 32A construct, 95 °C for 2 minutes, followed by 30 cycles of 95 °C for 30

seconds, 55 °C for 20 seconds, and 72 °C for 2 minutes, with a final extension of 72 °C for 10 minutes. The 32A construct containing **S**:**B** pairs was PCR amplified under conversion conditions with a small amount of d**B**TP, as described in the principal publication. The conversion product was directly cloned and sequenced.

(b) Nucleotide electrophoresis/gel extraction: Primary PCR products as well as secondary PCR amplicons of 16-fragment L-DNA assemblies were analyzed by agarose gel electrophoresis in TAE or TBE buffer (100 V for 20 min (50 V for 60 min for a gel extraction)). In the 32A and 32B constructs, the expected sized bands were cut and transferred to microcentrifuge tubes by shadow visualization under long wave UV (blue light for a gel extraction). A gel extraction was performed by using Zymoclean™ Gel DNA Recovery Kit.

(c) Sanger sequencing: Either purified (QiaQuick from Qiagen) primary PCR amplicon (32C), secondary PCR product from a ligation of gel-purified PCR amplified sub-assemblies (32B; see below for more detail on secondary ligation), or cloned, PCR converted (**S**:**B** to T:A) full-length construct (32A) was sequenced (Big Dye v 3.1, Life Technologies) as per vendor instructions and analyzed via capillary-based automated DNA sequencing at an offsite facility (the Interdisciplinary Center for Biotechnology Research (ICBR) of University of Florida, Gainesville, FL, USA).

PCR and ligation of sub-assemblies (32B construct): Since PCR amplification failed to detect any product from autonomous self-assembly of all 32 fragments together of the 32B construct (**Figure 8** of manuscript), the products of the two 16-fragment sub-assemblies were each PCR amplified, using 50 µL of the same reaction mixture and 0.5 µL of the primary PCR products. A reaction mixture (20 µL, 1X T4 DNA Ligase Buffer, 20 U/mL

T4 DNA Ligase (New England Biolabs) with added oligonucleotides (gel extracted 16-fragment assemblies, 50 ng each) was prepared in a microcentrifuge tube on ice. The reaction was incubated for two hours at room temperature and the completed ligation reaction of 32-fragment assemblies was PCR amplified. Amplicons were loaded on an agarose gel (1%) and separated in TAE buffer with 100 V for 30 minutes (**Figure S4**).

Results and Conclusions

When AEGIS nucleotides were used to assist annealing, a full-length product was obtained in the first try by PCR amplification of the AEL construct (**Figure 7** of manuscript). This was not necessarily the case when comparable attempts were made from the constructs using only standard nucleotides (32B and 32C) as discussed below (manuscript **Figure 8**, **Figure S4** and **Figure S5**).

No full-length AEL product was observed when all 32 fragments of the 32B set were mixed (last lane, **Figure 8**). To rule out the possibility that the oligonucleotides were defective, smaller constructs were self-assembled. **Figure 8** shows the results of stepwise assembly of sub-sets of the fragments, after the target ligation products are rescued from the mixture by PCR (30 cycles). Attempts to assemble 20, 24, 28, and 32 fragments failed to yield any detectable amplicon. Products arising from self-assembly of 4, 8 and 16 could be recovered by PCR in decreasing yields (**Figure 8**).

An alternate strategy was to independently assemble and PCR amplify the two halves (oligos #1 to 16 and oligos #17 to 32) of the 32B construct. This created half assemblies in large amounts, which then could be ligated with blunt ends. The desired 1135 base pair target construct was then recovered by PCR (**Figure S4**). This process, which represents the same

stepwise convergent assembly of L-DNA that has been used previously [3-5][6], of course, is not automated.

Autonomous self-assembly of multiple single stranded fragments can fail for many "trivial" reasons. Simplest among these is the fact that single stranded folding (e.g., to give hairpins) can compete with intermolecular hybridization (**Figure 1**). Hairpin formation (**Figure S6**) may have contributed to the failure of the 32B assemblies involving 20 or more oligonucleotides (**Figure 8 o**f principal manuscript). Since single stranded hybridization is a unimolecular process, the rate of folding and the corresponding equilibrium constant are independent of the concentration of the oligonucleotide. Thus, it competes more effectively with desired bimolecular hybridizations when the concentrations of the DNA fragments are low, an easy outcome when attempting to autonomously assembly many fragments.

Hairpins with short stems are, of course, impossible to avoid. For example, the 3′-end of a standard oligonucleotide must be G, A, C, or T; it must therefore find a partner with a 25% probability to form a hairpin having a loop of any arbitrary length with a single base pair in the stem. Likewise, any dinucleotide has a 6% probability of forming a hairpin having a loop of any arbitrary length with a two base pair stem.

The 32C assembly attempt also failed at first. Successful self-assembly of 32 fragments built from standard nucleotides was identified only once; after multiple tries and only after increasing the AEL concentrations of oligonucleotide fragments from 62.5 nM to 125 nM could a PCR product of the desired length be recovered (**Figure S5)**. While general conclusions are difficult to draw from these experiments alone, it appears that addition of AEGIS nucleotides to procedures that synthesize L-DNA constructs advances further the performance of automated and semi-automated gene synthesis.

Table S1: Selected sequences of kanamycin resistance gene assembled using AEGIS S:B pairs obtained from *E. coli* displaying resistance to kanamycin

* indicates a site where none of the sequences displayed an error

```
KanR_AEGIS                  CTAGTGGSCGBTCTGSCCGTCCTGTCAGCTGCTBGSCGSGCGGATCCTG 50
KanR_normal                 ------------------------------------------------- 1
Kan09 with dBTP             --------------GTCCGACCTGTCAGCTGCTAGTCGTGCGGATCCTG 36
Kan11 with dBTP             --------------GTCCGTCCTGTCAGCTGCTAGTCGTGCGGATCCCG 36
Kan14 with dBTP             ------------------GTCCTGCCAGCTGCTATTCGGGGGGATCCTG 32

KanR_AEGIS                TTAGAAAAACTCATCGAGCATCAAATGAAACTGCAASTTBTTCATBTCBGG 100
KanR_normal               TTAGAAAAACTCATCGAGCATCAAATGAAACTGCAATTTATTCATATCAGG 51
Kan09 with dBTP           TCAGAAAAACTCATCGAGCATCAAATGAAACTGCAATTTGTTCATATCCGG 86
Kan11 with dBTP           TTAGAAAAACTCATCGAGCATCAAATGAAACTGCAATTTATTCATATCAGG 86
Kan14 with dBTP           TTAGATCC-TTCATCGAGCATCATATGAAACTGCAATTTATTCATATCAGG 81
                          *****     ************* *************** ******** **

KanR_AEGIS                 ATTATCAATACCATATTTTTGAAAAAGCCGSTTCTGTAASGABGGAGAAA 150
KanR_normal                ATTATCAATACCATATTTTTGAAAAAGCCGTTTCTGTAATGAAGGAGAAA 101
Kan09 with dBTP            ATTATCAATACCATATTTTTGAAAAAGCCGTTTCTGTAATGAAGGACAAA 136
Kan11 with dBTP            ATTATCAATACCATATTTTTGAAAAAGCCGTTTCTGTAATGAAGGAGAAA 136
Kan14 with dBTP            ATTATCAATACCATATTTTTGAAAAAGCTTTTTCTGCAATGACCGAAAAA 131
Kan12 without dBTP         ATTATCAATACCATATTTTTGAAAAAGCCGTTTCTGTAATGAAGGAGAAA 136
Kan13 without dBTP         ATTATCAATACCATATTTTTGAAAAAGCCGCTTCTGTAATGAAGGAGAAA 136
                           ****************************   ***** *****  ** ***

KanR_AEGIS                 ACTCACCGAGGCAGTTCCATAGGATGGCBAGBTCCTGGTASCGGTCTGCG 200
KanR_normal                ACTCACCGAGGCAGTTCCATAGGATGGCAAGATCCTGGTATCGGTCTGCG 151
Kan09 with dBTP            ACTCACCGAGGCAGTTCCATAGGATGGCAAGATCCTGGTATCGGTCTGCG 186
Kan11 with dBTP            ACTCACCGAGGCAGTTCCATAGGATGGCAAGATCCTGGTATCGGTCTGCG 186
Kan14 with dBTP            ACTCACCGAGGCAGTTCCATAGGATGGCAAGATCCTGGTATCGGTCTGCG 181
                           **************************************************

KanR_AEGIS                 ATTCCGACSCGSCCBACBTCAATACAACCTATTAATTTCCCCTCGTCAAA 250
KanR_normal                ATTCCGACTCGTCCAACATCAATACAACCTATTAATTTCCCCTCGTCAAA 201
Kan09 with dBTP            ATTCCGACTCGTCCAACATCAATACAACCTATTAATTTCCCCTCGTCAAA 236
Kan11 with dBTP            ATTCCGACTCGTCCAACATCAATACAACCTATTAATTTCCCCTCGTCAAA 236
Kan14 with dBTP            ATTCCGACTCGTCCAACATCAATACAACCTATTA-TTTCCCCTCGTCAAA 230
                           ********************************** ***************

KanR_AEGIS                 AATAAGGTTBTCBAGSGAGAABTCACCATGAGTGACGACTGAATCCGGTG 300
KanR_normal                AATAAGGTTATCAAGTGAGAAATCACCATGAGTGACGACTGAATCCGGTG 251
Kan09 with dBTP            AATAAGGTTATCAAGTGAGAAATCACCATGAGTGACGACTGAATCCCGTG 286
Kan11 with dBTP            AATAAGGTTATCAAGTGAGAAATCACCATGAGTGACGACTGAATCCGGTG 286
Kan14 with dBTP            AATAAGGTTATCAAGAGAGAAATCTCCATGAGTGACGACTGAATTTTGTA 280
                           ************************ *******************   **

KanR_AEGIS                 AGAASGGCAABAGSTTBTGCATTTCTTTCCAGACSTGSTCBACBGGCCAG 350
KanR_normal                AGAATGGCAAAAGTTTATGCATTTCTTTCCAGACTTGTTCAACAGGCCAG 301
Kan09 with dBTP            AGAATGGCAAAAGTTTATGCATTTCTTTCCAGACTTGTTCAACAGGCCAG 336
Kan11 with dBTP            AGAATGGCAAAAGTTTATGCATTTCTTTCCAGACTTGTTCAACAGGCCAG 336
Kan14 with dBTP            AGAATGGCAAAAGTTTATGCATTTCTTTCCAGACTTGATCAACAGGCCAG 330
                           **************************************************

KanR_AEGIS                 CCATTACGCTCGTCATCAAAATCACTCGCBTCBACCAABCCGTTATTCAT 400
KanR_normal                CCATTACGCTCGTCATCAAAATCACTCGCATCAACCAAACCGTTATTCAT 351
Kan09 with dBTP            CCATTACGCTCGTCATCAAAATCACTCGCATCAACCAACCCGTTATTCAT 386
Kan11 with dBTP            CCATTACGCTCGTCATCAAAATCACTCGCATCAACCAAACCGTTATTCAT 386
Kan14 with dBTP            CCATTACGCTCGTCATCAAAATCACTCGCATCAACCAACCCGTTATTCAT 380
                           ******************************** ***** ***********

KanR_AEGIS                 TCGTGATTGCGCCTGBGCGAGBCGAAATACGCGATCGCTGTTAAAAGGAC 450
KanR_normal                TCGTGATTGCGCCTGAGCGAGACGAAATACGCGATCGCTGTTAAAAGGAC 401
Kan09 with dBTP            TCGTGATTGCGCCTGAGCGAGACGAAATACGCGATCGCTGTTAAAAGGAC 436
Kan11 with dBTP            TCGTGATTGCGCCCGAGCGAGACGAAATACGCGATCGCTGTTAAAAGGAC 436
Kan14 with dBTP            TCGTGATTGCGCCTGAGCGAGACGAAATACGCGATCGCTGTTAAAAGGAC 430
                           ************* ************************************

KanR_AEGIS                 AATTACBAACBGGAATCGABTGCAACCGGCGCAGGAACACTGCCAGCGCA 500
KanR_normal                AATTACAAACAGGAATCGAATGCAACCGGCGCAGGAACACTGCCAGCGCA 451
Kan09 with dBTP            AATTACAAACAGGAATCGAATGCAACCGGCGCAGGAACACTGCCAGCGCA 486
Kan11 with dBTP            AATTACAAACAGGAATCGAATGCAACCGGCGCAGGAACACTGCCAGCGCA 486
Kan14 with dBTP            AATTACAAACAGGAATCGAATGCAACCGGCGCAGGAACACTGCCAGCGCA 480
```

```
                          **************************************************

KanR_AEGIS                TCBACAATBTTSTCBCCTGAATCAGGATATTCTTCTAATACCTGGAASGC 550
KanR_normal               TCAACAATATTTTCACCTGAATCAGGATATTCTTCTAATACCTGGAATGC 501
Kan09 with dBTP           TCAACAATATTTTCACCTGAATCAGGATATTCTTCTAATACCTGGAATGC 536
Kan11 with dBTP           TCAACAATATTTTCACCTGAATCAGGATATTCTTCTAATACCTGGAATGC 536
Kan14 with dBTP           TCAACAATATTTTCACCTGAATCAGGATATTCTTCTAATACCTGGAATGC 530
                          **************************************************

KanR_AEGIS                SGTSTTSCCGGGGATCGCAGTGGTGAGTAACCATGCATCBTCBGGBGTBC 600
KanR_normal               TGTTTTTCCGGGGATCGCAGTGGTGAGTAACCATGCATCATCAGGAGTAC 551
Kan09 with dBTP           TGTTTTTCCGGGGATCGCAGTGGTGAGTAACCATGCATCATCAGGAGTAC 586
Kan11 with dBTP           TGTTTTTCCGGGGATCGCAGTGGTGAGTAACCATGCATCATCAGGAGTAC 586
Kan14 with dBTP           TGTTTTTCCGGGGATCGCAGTGGTGAGTAACCATGCATCATCAGGAGTAC 580
                          **************************************************

KanR_AEGIS                GGATAAAATGCTTGATGGTCGGBAGBGGCATAAASTCCGTCAGCCAGTTT 649
KanR_normal               GGATAAAATGCTTGATGGTCGGAAGAGGCATAAATTCCGTCAGCCAGTTT 600
Kan09 with dBTP           GGATAAAATGCTTGATGGTCGGAAGAGGCATAAATTCCGTCAGCCAGTTT 635
Kan11 with dBTP           GGATAAAATGCTTGATGGTCGGAAGAGGCATAAATTCCGTCAGCCAGTTT 635
Kan14 with dBTP           GGATAAAATGCTTGATGGTCGGAAGAGGCATAAATTCCGTCAGCCAGTTT 629
                          **************************************************

KanR_AEGIS                AGTCTGACCATCTCATCTGTBACBTCBTTGGCABCGCTACCTTTGCCATG 698
KanR_normal               AGTCTGACCATCTCATCTGTAACATCATTGGCAACGCTACCTTTGCCATG 649
Kan09 with dBTP           AGTCTGACCATCTCATCTGTAACATCATTGGCAACGCTACCTTTGCCATG 684
Kan11 with dBTP           AGTCTGACCATCTCATCTGTCACATCATTGGCAACGCTACCTTTGCCATG 684
Kan14 with dBTP           AGTCTGACCATCTCATCTGTAACATCATTGGCCACGCTACCTTTGCCATG 678
                          ******************************** *****************

KanR_AEGIS                TTTCAGAAACAACTCSGGCGCBTCGGGCTTCCCATACAAGCGATAGATTG 744
KanR_normal               TTTCAGAAACAACTCTGGCGCATCGGGCTTCCCATACAAGCGATAGATTG 695
Kan09 with dBTP           TTTCAGAAACAACTCGGGCGCATCGGGCTTCCCATACAAGCGATAGATTG 730
Kan11 with dBTP           TTTCAGAAACAACTCTGGCGCATCGGGCTTCCCATACAAGCGATAGATTG 730
Kan14 with dBTP           TTTCAGAAACAACTCTGGCGCATCGGGCTTCCCATACAAGCGATAGATTG 724
                          *************** ***** ****************************

KanR_AEGIS                TCGCACCSGASTGCCCGACBTTATCGCGAGCCCATTTATACCCATATAAB 786
KanR_normal               TCGCACCTGATTGCCCGACATTATCGCGAGCCCATTTATACCCATATAAA 737
Kan09 with dBTP           TCGCACCTGATTGCCCGACATTATCGCAAGCCCATTTATACCCATATAAA 772
Kan11 with dBTP           TCGCACCTGATTGCCCGACATTATCGCGAGCCCATTTATACCCATATAAA 772
Kan14 with dBTP           TCGCACCTGATTGCCCGACATTATCGCGAGCCCATTTATACCCATATAAA 766
                          *************************** **********************

KanR_AEGIS                TCBGCBTCCATGTTGGAATTTAATCGCGGCCTCGACGTTTCCCGTTGAAT 827
KanR_normal               TCAGCATCCATGTTGGAATTTAATCGCGGCCTCGACGTTTCCCGTTGAAT 778
Kan09 with dBTP           TCAGCATCCATGTTGGAATTTAATCGCGGCCTCGACGTTTCCCGTTGAAT 813
Kan11 with dBTP           TCAGCCTCCATGTTGGAATTTAATCGCGGCCTCGACGTTTCCCGTTGAAT 813
Kan14 with dBTP           TCAGCATCCATGTTGGAATTTAATCGCGGCCTCGACGTTTCCCGTTGAAT 807
                          ***** ********************************************

KanR_AEGIS                ATGGCTCATGGTG
KanR_normal               ATGGCTCAT----
Kan09 with dBTP           ATGGCTCATGGTG
Kan11 with dBTP           ATGGCTCATGGTG
Kan14 with dBTP           ATGGCTCATGGTG
                          *********
```

Representative sequences of the antisense strand of various gene encoding kanamycin resistance, determined by classical Sanger sequencing from a plasmid prepped from transformed cells grown in the presence of kanamycin. The PCR primer is underlined, not bold. The start sequence in the gene (**CAT**, antisense) is bold underlined. Top line shows the putative construct, including the **S** and **B** nucleotides used to provide controlled orthogonal assembly of the ML-DNA construct. The second line shows the sequence of the native gene encoding the kanamycin resistance protein. The conversion that generated these sequences was done with a small amount of dBTP, requiring only that dTTP mismatch dB in the template. The sequencing results have the expected features, in particular, the loss of quality towards the end of the read. These results show that conversion of S to T and B to A was no less faithful than the sequences obtained generally, which include PCR and sequencing error.

Table S2: Master Mix for conversion PCR

Item	Per reaction	Master Mix (x5)
Taq Full Buffer, 10x	5 µL	25 µL
dNTP (stock 10 mM)	1 µL	5 µL
KanR_For primer (stock 10 µM)*	2 µL	10 µL
KanR_Rev primer (stock 10 µM)*	2 µL	10 µL
Taq Full polymerase	0.4 µL	2.0 µL
Water	37.6 µL	188 µL
Template (DNA or water)	2 µL	------
disoGTP (dB)	0.3 or 0 µL	-----
Total Volume	50 or 50.3 µL	

*KanR For: CACCATGAGCCATATTCAACGG
*KanR Rev: GTCCGTCCTGTCAGCTGC

Table S3. Secondary PCR recipe and setup

Item	Per reaction	Master Mix (x4)
5X PrimeSTAR GXL	10 µL	40 µL
dNTP (10 mM)	1 µL	4 µL
KanR_For primer (10 µM)	1.5 µL	6 µL
KanR_Rev primer (10 µM)	1.5 µL	6 µL
PrimeSTAR polymerase	1 µL	4 µL
Water	34 µL	136 µL
Template (DNA or water)	1 µL	------

Analysis of all sequences of kanamycin resistance gene assembled using AEGIS S:B pairs

Sequences obtained:

Table S4 summarizes the sequences obtained from a series of plasmid preps obtained from *E. coli* before selecting for kanamycin resistance. The "status" was determined by looking at both the upstream and downstream sequencing for a submission and determining if the entire gene was present (Full), if no gene was present (Missing), if there was an incomplete assembly (Incomplete), or if the status could not be determined (?), the last arising from failure in either the upstream or downstream sequencing.

Table S4: Analysis of *E. coli* plasmid prep sequences without selection for resistance

Query	Q. Start	Q. End	S. Start	S. End	Strand	Q. Size	Length	Status
KRplus20_T7Term.ab1	140	986	850	1	Minus	1066	847	Full
KRplus20_T7Long.ab1	77	924	1	850	Plus	1237	848	Full
KRplus19_T7Term.ab1	141	799	850	192	Minus	799	659	Full
KRplus19_T7Long.ab1	80	917	1	839	Plus	920	838	Full
KRplus18_T7Term.ab1	140	637	850	352	Minus	637	498	?
KRplus17_T7Term.ab1	163	645	1	482	Plus	645	483	Full
KRplus17_T7Long.ab1	79	839	850	91	Minus	842	761	Full

KRplus15_T7Term.ab1	141	878	850	115	Minus	881	738	Full
KRplus15_T7Long.ab1	60	912	1	850	Plus	1128	853	Full
KRplus14_T7Term.ab1	141	993	850	1	Minus	997	853	Full
KRplus14_T7Long.ab1	78	929	1	851	Plus	995	852	Full
KRplus13_T7Term.ab1	143	675	1	534	Plus	680	533	Full
KRplus13_T7Long.ab1	75	923	850	1	Minus	1230	849	Full
KRplus12_T7Term.ab1	141	920	850	73	Minus	920	780	Full
KRplus12_T7Long.ab1	63	911	1	850	Plus	1155	849	Full
KRplus11_T7Long.ab1	61	709	1	650	Plus	716	649	?
KRplus10_T7Term.ab1							1	Missing
KRplus10_T7Long.ab1							0	Missing
KRplus09_T7Long.ab1							0	Missing
KRplus08_T7Term.ab1	142	989	850	4	Minus	1069	848	Full
KRplus08_T7Long.ab1	75	924	1	850	Plus	1164	850	Full
KRplus07_T7Term.ab1	162	1016	857	4	Minus	1062	855	Full
KRplus07_T7Long.ab1	63	880	1	815	Plus	880	818	Full
KRplus06_T7Term.ab1	139	755	850	228	Minus	755	617	?
KRplus05_T7Long.ab1							0	Missing
KRplus04_T7Term.ab1	141	594	1	454	Plus	597	454	Full
KRplus04_T7Long.ab1	76	876	850	48	Minus	878	801	Full
KRplus03_T7Term	141	639	850	352	Minus	639	499	Full
KRplus03_T7Long.ab1	77	877	1	802	Plus	879	801	Full
KRplus02_T7Term	139	687	850	303	Minus	687	549	Full
KRplus02_T7Long	96	594	1	500	Plus	594	499	Full
KRplus01_T7Term	140	624	1	486	Plus	624	485	Full
KRplus01_T7Long.ab1	74	922	850	1	Minus	1151	849	Full
KRmin_19_T7Term.ab1	141	951	850	42	Minus	957	811	Full
KRmin_19_T7Long.ab1	63	913	1	850	Plus	1031	851	Full
KRmin_18_T7Term.ab1	140	555	1	415	Plus	555	416	Incomplete
KRmin_18_T7Long.ab1	76	601	526	1	Minus	639	526	Incomplete
KRmin_17_T7Long.ab1	78	681	850	247	Minus	681	604	?
KRmin_16_T7Long.ab1							0	Missing
KRmin_15_T7Term.ab1	156	679	526	1	Minus	760	524	Incomplete
KRmin_13_T7Term.ab							0	Missing

1								
KRmin_13_T7Long.ab1							0	Missing
KRmin_12_T7Term.ab1	142	667	1	526	Plus	798	526	Incomplete
KRmin_12_T7Long.ab1	79	604	526	1	Minus	881	526	Incomplete
KRmin_11_T7Term.ab1	141	665	1	526	Plus	757	525	Incomplete
KRmin_11_T7Long.ab1	77	601	526	1	Minus	847	525	Incomplete
KRmin_10_T7Term.ab1							0	Missing
KRmin_09_T7Term.ab1	140	986	850	1	Minus	994	847	Full
KRmin_09_T7Long.ab1	85	803	1	716	Plus	803	719	Full
KRmin_08_T7Term.ab1	141	666	1	526	Plus	676	526	Incomplete
KRmin_08_T7Long.ab1	77	602	526	1	Minus	1234	526	Incomplete
KRmin_06_T7Term.ab1	145	641	1	498	Plus	641	497	Incomplete
KRmin_06_T7Long.ab1	76	598	526	1	Minus	915	523	Incomplete
KRmin_05_T7Term.ab1	143	957	1	815	Plus	957	815	Full
KRmin_05_T7Long.ab1	77	930	850	1	Minus	965	854	Full
KRmin_04_T7Term.ab1	143	760	1	616	Plus	763	618	Full
KRmin_04_T7Long.ab1	77	922	850	4	Minus	1025	846	Full
KRmin_03_T7Term.ab1							0	Missing
KRmin_03_T7Long.ab1							0	Missing
KRmin_02_T7Term.ab1	142	665	1	526	Plus	801	524	Incomplete
KRmin_02_T7Long.ab1	76	599	526	1	Minus	915	524	Incomplete
KRmin_01_T7Term.ab1							0	Missing
KRmin_01_T7Long.ab1							0	Missing

As a breakdown of the above information, the counts for each category of gene completeness are shown in Table S5.

Table S5: Summary of completeness in self-assembled kanamycin resistance gene

Gene Status	Count
Full	17
Incomplete Assembly	7
Missing	8
Unknown (?)	4

No incomplete assemblies were found when d**B**TP was used in the conversion PCR; 13 full assemblies were found under these conditions. This can be compared to 7 incomplete assemblies found when d**B**TP was absent in the conversion PCR; here, only 4 full assemblies.

Error in self-assembled kanamycin resistance gene:

Table S6 compares errors in this set of sequencing results to a set of 31 sequences obtained from cultured *E. coli* shown to have resistance to kanamycin (full data not shown; selection shown in **Table S1**). This comparison shows no appreciable increase in errors due to conversion between sequences conferring kanamycin resistance and all amplified sequences. Locations that underwent conversion from AEGIS bases had slightly higher error rates than those that did not. The overall rate of error is also much lower in this set of sequences as compared to the kanamycin-positive set, likely due to overall cleaner sequencing run. These data show slightly more conversion errors when d**B**TP was used in the conversion PCR (47 errors) compared with when d**B**TP was absent in the conversion PCR (30).

Table S6: Comparison of sequences between selection/no selection data sets

	KanR Positive	KanR All
Non-Conversion Error Rate	2.0%	0.8%
S Conversion Total Error Rate	4.6%	3.5%
S Conversion S->C Errors	0.9%	1.1%
S Conversion S->Other Errors	3.7%	2.4%
B Conversion Total Error	5.4%	2.2%
B Conversion B->G Errors	1.8%	0.4%
B Conversion B->Other Errors	3.6%	1.8%

Table S7: Fragment sequences in the 32A construct

Order	Oligonucleotide	Strand
1	GCBTTGCGSCCATCBAGCAGTGGCTGTATACCGGABGTGGGSCGGCTCST	Minus
2	SGATGGBCGCAASGCTGTTTACTCGGTCAGTAGAGGGCGBACGABTGTBG	Plus
3	STGTCBCCTGSCCGCTTCAAAACCCTTCATTCCTACSACASTCGTSCGCC	Minus
4	GCGGBCAGGSGACABAGAATACTCTATAGGATCACBCGCTBTCAGGGTST	Plus
5	CABCCCGTSCGTABGTATCGATTTCCTTGGCATABACCCTGASAGCGSGT	Minus
6	CSTACGBACGGGSTGAGAACTGTGAAAACAACCGTSAGGTGCSGGGTSGG	Plus
7	CCBCGGCBTCCTABGTGTATACCAATAGGTCCAGTCCBACCCBGCACCTB	Minus
8	CSTAGGASGCCGSGGATAAGAGATGTTCCCTAGACSTCAGACBGGACBCT	Plus
9	GSGTBGGCBCGCGTBTGTTACTCACAACTAATGAGSGTCCSGTCTGABGT	Minus
10	SACGCGSGCCSACBCAACTACGTAGTGACATGCTABTCTCCSGCTCGCCB	Plus
11	CGSGCSCGGBATBCCTTTACATCAGTTCCGCATCTSGGCGAGCBGGAGAS	Minus
12	GGSATSCCGBGCBCGTTTAGTCTTCTAACACAGASGTCGCTASCTCBCGT	Plus
13	BCGTCGTCSAGCSCCAGAGGAGAGAGAAAGTTTACGSGAGBTAGCGACBT	Minus
14	GGBGCTBGACGACGSATACAATACCCACTATGGTCSGGGAASGGGGSCCC	Plus
15	CGBATGTBCGCTCBCATTGATGATATGCCTCAACAGGGBCCCCBTTCCCB	Minus
16	GSGAGCGSACATSCGACTTTTCATGTATCTATAACGSTACGBCGTCCSAT	Plus
17	BCCTCATCBCSGCGGCTAAGATCGTGAGCTAATATBGGACGSCGTABCGT	Minus
18	CCGCBGSGATGAGGSAATAGTCGTGTTGTAGAGAACCBCTCABGGACBCG	Plus
19	CGCSABGCCCBGGTBTCAAGAAGAAGTCTTATGGGCGSGTCCSTGAGSGG	Minus
20	SACCSGGGCSTBGCGTAGAAATGTTTTGCTTAAABASGCCTAGBGGGCBT	Plus
21	STCGGCABGGGAAGSCCAGTTTTGTAGCTAACTASGCCCSCTAGGCBTST	Minus
22	BCTTCCCSTGCCGABATTAGCGACTTAAGGATAACCGCGSABTGGASGGC	Plus
23	BGCCBAAGAGCCBCBAGTCGGTGCATTTGTCTTAGGCCBTCCASTBCGCG	Minus
24	SGSGGCTCTTSGGCSATTCATCTATAGAACTTGACBGGBGCGSGTASGGT	Plus
25	GGSACGBCAASGGGCAGCCGTATCTTCTGTATTACCBTACBCGCSCCSGT	Minus
26	GCCCBTTGSCGTBCCAGTATCCATTCCATACGTTGGBAACCABTCCGGSG	Plus
27	SCCGGBCGGBTTCCBTATACCCTTTCATATGATGCCBCCGGASTGGTTSC	Minus
28	SGGAASCCGSCCGGBTATAGGTTTAGATGTTAGASTCGGTCSGCSAGSGT	Plus
29	SGGACTCCBCGASCCTAGTACAATGTTACATTGACBCTBGCBGACCGABT	Minus
30	GGBTCGSGGAGTCCBAAATGGAATAGTAGAGCATCCGCGBGSTCATTCSC	Plus
31	CSGTGGGGTBGCACBTTATGATGGTGAAATGTTTAGBGAATGABCSCGCG	Minus
32	SGTGCSACCCCACBGTGAAAAGTAGACGATCTAABTGTTGCBAGCGCSCT	Plus

Table S8: Fragment Sequences in the 32B construct

Order	Oligonucleotide	Strand
1	CTGTCGGATCCCGCTTGGATGTGTACGCTTGGGGTAGCTGGGAGGCTCTT	Minus
2	AGCGGGATCCGACAGGGTTGACGATTACAAAGGCAGGAGGGCATCAACTG	Plus
3	CCGGTGAGCTCCTCAGGATGGGTTAAGAAACAAAACAGTTGATGCCCTCC	Minus
4	TGAGGAGCTCACCGGATCAATACATGACGAAGTAGCCGATTTGGAGTGTT	Plus
5	CTATCGCCTCGGCATATGATTCTACATTTGACAAAAACACTCCAAATCGGCT	Minus
6	ATGCCGAGGCGATAGCATTCTTTTTAAACACCTTTAGCCGAACTATGGCC	Plus
7	GTGCAAGGCCTGATTACCATTGATACTTCACTTCTGGCCATAGTTCGGCT	Minus
8	AATCAGGCCTTGCACTTGCTACATTACTTTTCTAGACAAAGAGACGGGTT	Plus
9	GCAATGACGGACTTGAAACCATAACTAGCTCGAGTAACCCGTCTCTTTGTCT	Minus
10	CAAGTCCGTCATTGCTTGAAGGACCGAATTCATTAGCCGATAGGTACGTC	Plus
11	ACGAACGAGCCGTTATTCTATAGAGCTCGTGAGACGACGTACCTATCGGC	Minus
12	TAACGGCTCGTTCGTATAACAATACACTTTCACACGTTCTTCAGTGACGT	Plus
13	CGCAAAGAGCGACAGAAGCAACGTGGATAAGCTCTACGTCACTGAAGAACGT	Minus
14	CTGTCGCTCTTTGCGAAAGTAAGTTAACATGTTTGGACCACTGCCAGTAC	Plus
15	CGCTCTTCCTGGCTAGTCATCTGTGGGTATTCCTCGTACTGGCAGTGGTC	Minus
16	TAGCCAGGAAGAGCGATTACGGAAAGGTCAAAAATCTTTCCAGGGCACGT	Plus
17	GGGTCATCCCTACGGTGTTATCTTTTCCGCTGATAACGTGCCCTGGAAAGAT	Minus
18	CCGTAGGGATGACCCTCTAGAAGTCGAGGGGTAGTAGCTAGGCCACAGAC	Plus
19	ACCAGGACGTCTGGATCTAAGTATGTCTCTAAGCAGTCTGTGGCCTAGCT	Minus
20	TCCAGACGTCCTGGTCTTAGAGAACATATGTAAACGACGTGTACCGTTCT	Plus
21	CAGCGTGAGGCCAATTTAGTTACTCATTCCCCAGTAGAACGGTACACGTCGT	Minus
22	ATTGGCCTCACGCTGATTGGTCTTATCAGACGCTGGGCGTTTAAACCGGT	Plus
23	ACGCCACTTACGCCAGCGATAAAGGCCTACTCAACACCGGTTTAAACGCC	Minus
24	TGGCGTAAGTGGCGTGCTGAAGTCCTATAGTTTAGGAAGCAACAGCATGT	Plus
25	CGTCAGTTAACCGCAACATTGAGTATTCGCCTGAAACATGCTGTTGCTTCCT	Minus
26	TGCGGTTAACTGACGACAAGCATTACATTCACCATAAATGCCACAGGACG	Plus
27	GCTTCCTTCTCAGCCTCCGACTCTAGTTCATAGTACGTCCTGTGGCATTT	Minus
28	GGCTGAGAAGGAAGCGGTATACTCTGTTTTCTTATAGTTCCGACCGACGT	Plus
29	GAGCGGAAGTGTGCTTAGTAATGACGTCAACCTATACGTCGGTCGGAACTAT	Minus
30	AGCACACTTCCGCTCTCTTTCTGAGTATGGTCCTTAAGACTGGGCACAAC	Plus
31	CTCTTGGATCCACCGCACCTGTGTACTACTTCTCTGTTGTGCCCAGTCTT	Minus
32	CGGTGGATCCAAGAGATTACACTGGCTTTACCCAAGGATAGTACGCGAGT	Plus

Table S9: Fragment sequences in the 32C construct

Order	Oligonucleotide	Strand
1	TGCTTGGATCCCCTCCCTCTATGAAGAGACCTCGTATGGCGTTGCACTGT	Minus
2	GAGGGGATCCAAGCAATCCGCAGTAAGCTGTCAAATATCCCCACCACCAC	Plus
3	CTGAGGACGTCGCATTAGCTGAAGCCTTACGGATAGTGGTGGTGGGGATA	Minus
4	ATGCGACGTCCTCAGATTGTGCGCTCTTTCCGCAAGCCCACTAAAGACCT	Plus
5	CCCTAGTTCGGGACACCGTATCTAAACTTTCTAACAGGTCTTTAGTGGGCTT	Minus
6	TGTCCCGAACTAGGGGGAGTTAGAGCTCTGATAACCAGTGGCCTGTTTTG	Plus
7	GCTCGTTTAAACCGCTAGTGTAGCATGGTCAATTCCAAAACAGGCCACTG	Minus
8	GCGGTTTAAACGAGCAGAATTGACTTCTAAACGATGGAGCACAGGGTCAT	Plus
9	GACACATGGGCTTGTCATAACATCAACTCATTCTTATGACCCTGTGCTCCAT	Minus
10	ACAAGCCCATGTGTCGTAGCTATAGGTGTAAGTGCGCAACGTATGGTACG	Plus
11	TTGCGTCCACGTTTGTAGACCAGACGTCCGTACTTCGTACCATACGTTGC	Minus
12	CAAACGTGGACGCAAAAATCTCTAGGGCTAACCATTACACGTGAACCCGT	Plus
13	GTCACCCGTGCTGTAAAGCAAATCTTTGGGGATATACGGGTTCACGTGTAAT	Minus
14	TACAGCACGGGTGACACTTAACAGGCCTAAACTCTGCAGGAACTTTGCTC	Plus
15	GGGCTACGAAGTCGATAGAAGGACTACACCTGCCAGAGCAAAGTTCCTGC	Minus
16	TCGACTTCGTAGCCCAAAGCACATATCCAATAGAAACCATTTGCGAAGGT	Plus
17	TGGCCTCGTGCGTATATGAGTATCATTGATCTTTGACCTTCGCAAATGGTTT	Minus
18	ATACGCACGAGGCCAACCATAACCTAAACGGCTATGGCAAACGCGACTCA	Plus
19	GCGAGGTTAACGCTTTGCCGAGTCACTAGCAATACTGAGTCGCGTTTGCC	Minus
20	AAGCGTTAACCTCGCGAAGAGATAAGCAGATATACACGGTATAGTGCCTT	Plus
21	CACTTCAGGCTGTCGCTTCGAATGACAGGATAGTAAAGGCACTATACCGTGT	Minus
22	CGACAGCCTGAAGTGAGATATGGGTGAATTGATTAAGGGGAGCTCGACGT	Plus
23	GTTTGGACGAATGGGATATCACTTTAAACCGACACACGTCGAGCTCCCCT	Minus
24	CCCATTCGTCCAAACGCAGGATTTCCTTTGTGTATTCCGTGGGACCACAT	Plus
25	CCTGAAGGCCTACCTGGTTGAAACCCTAACTGCTGATGTGGTCCCACGGAAT	Minus
26	AGGTAGGCCTTCAGGAGGGATATGTTCACACATTGGGACACGCGATAAGC	Plus
27	GGGCTCCGTTTTCTTGCAAAACTGGATCACCAGATGCTTATCGCGTGTCC	Minus
28	AAGAAAACGGAGCCCTTAGATGATGATGGAATTAAGAACCGCACATGAGT	Plus
29	CACGGTCAGTGTCTGATACTACGTTAACGACAATTACTCATGTGCGGTTCTT	Minus
30	CAGACACTGACCGTGACCATAAGATTAGATTACTATCCACCCTGCCCAAA	Plus
31	GCCACGGATCCTAGAAGAAATCCTATTGGCTGGAATTTGGGCAGGGTGGA	Minus
32	TCTAGGATCCGTGGCTAACAGGAATGATGTTTAACTTCACTCACCTCGAT	Plus

Table S10: Primer sequences to analyze the 32A construct

Order	Primer	Orientation
1	ABGAGCCSCCCBGCU	+
2	ABCACTCCBAASCGGCU	-
3	AGCCGBTTSGGAGTGSU	+
4	ABCCCGTCSCTTTGSCU	-
5	AGBCAAAGBGACGGGSU	+
6	ACGSCACSGAAGABCGU	-
7	ACGSTCTTCBGTGBCGU	+
8	ACGSGCCCSGGAAAGBU	-
9	ASCTTTCCBGGGCBCGU	+
10	AGBACGGTBCACGSCGU	-
11	ACGBCGTGSACCGTSCU	+
12	ACBTGCTGSTGCTSCCU	-
13	AGGBAGCABCAGCASGU	+
14	ACGSCGGSCGGAACTBU	-
15	ASAGTTCCGBCCGBCGU	+
16	ACSCGCGTACSATCCSU	-

Table S11: Primer sequences to analyze the 32B constructs and sub-constructs

Order	Primer	Orientation
1	AAGAGCCTCCCAGCT	+
2	AACACTCCAAATCGGCT	-
3	AGCCGATTTGGAGTGTT	+
4	AACCCGTCTCTTTGTCT	-
5	AGACAAAGAGACGGGTT	+
6	ACGTCACTGAAGAACGT	-
7	ACGTTCTTCAGTGACGT	+
8	ACGTGCCCTGGAAAGAT	-
9	ATCTTTCCAGGGCACGT	+
10	AGAACGGTACACGTCGT	-
11	ACGACGTGTACCGTTCT	+
12	ACATGCTGTTGCTTCCT	-
13	AGGAAGCAACAGCATGT	+
14	ACGTCGGTCGGAACTAT	-
15	ATAGTTCCGACCGACGT	+
16	ACTCGCGTACTATCCTT	-

Table S12: Primer sequences to analyze the 32C construct

Order	Primer	Orientation
1	ACAGTGCAACGCCAT	+
2	AGGTCTTTAGTGGGCTT	-
3	AAGCCCACTAAAGACCT	+
4	ATGACCCTGTGCTCCAT	-
5	ATGGAGCACAGGGTCAT	+
6	ACGGGTTCACGTGTAAT	-
7	ATTACACGTGAACCCGT	+
8	ACCTTCGCAAATGGTTT	-
9	AAACCATTTGCGAAGGT	+
10	AAGGCACTATACCGTGT	-
11	ACACGGTATAGTGCCTT	+
12	ATGTGGTCCCACGGAAT	-
13	ATTCCGTGGGACCACAT	+
14	ACTCATGTGCGGTTCTT	-
15	AAGAACCGCACATGAGT	+
16	ATCGAGGTGAGTGAAGT	-

```
Forward Fragments:                                            SGATGGBCGCAASGCTGTTTACTCGGTCAGTAGAGGGCGBACGAB
Reverse Fragments:         TSCTCGGCSGGGTGBAGGCCATATGTCGGTGACGABCTACCSGCGTTBCG                    CCGCSTGCTS
Assembled Construct: 1     ABGAGCCGBCCCACSTCCGGTATACAGCCACTGCTSGATGGBCGCAASGCTGTTTACTCGGTCAGTAGAGGGCGBACGAB 80

Forward Fragments:         TGTBG                    GCGGBCAGGSGACABAGAATACTCTATAGGATCACBCGCTBTCAGGGTST
Reverse Fragments:         ACASCATCCTTACTTCCCAAAACTTCGCCSGTCCBCTGTS                  TGSGCGASAGTCCCABATACGG
Assembled Construct: 81    TGTBGTAGGAATGAAGGGTTTTGAAGCGGBCAGGSGACABAGAATACTCTATAGGATCACBCGCTBTCAGGGTSTATGCC 160

Forward Fragments:                      CSTACGBACGGGSTGAGAACTGTGAAAACAACCGTSAGGTGCSGGGTSGG
Reverse Fragments:         TTCCTTTAGCTATGBATGCSTGCCCBAC                    BTCCACGBCCCABCCTGACCTGGATAACCATA
Assembled Construct: 161   AAGGAAATCGATACSTACGBACGGGSTGAGAACTGTGAAAACAACCGTSAGGTGCSGGGTSGGACTGGACCTATTGGTAT 240

Forward Fragments:            CSTAGGASGCCGSGGATAAGAGATGTTCCCTAGACSTCAGACBGGACBCT                  SACGCGSGC
Reverse Fragments:         TGTGBATCCTBCGGCBCC                  TGBAGTCTGSCCTGSGAGTAATCAACACTCATTGTBTGCGCBCG
Assembled Construct: 241   ACACSTAGGASGCCGSGGATAAGAGATGTTCCCTAGACSTCAGACBGGACBCTCATTAGTTGTGAGTAACASACGCGSGC 320

Forward Fragments:         CSACBCAACTACGTAGTGACATGCTABTCTCCSGCTCGCCB                    GGSATSCCGBGCBCGTTTA
Reverse Fragments:         GBTGSG                    SAGAGGBCGAGCGGSTCTACGCCTTGACTACATTTCCBTABGGCSCGSGC
Assembled Construct: 321   CSACBCAACTACGTAGTGACATGCTABTCTCCSGCTCGCCBAGATGCGGAACTGATGTAAAGGSATSCCGBGCBCGTTTA 400

Forward Fragments:         GTCTTCTAACACAGASGTCGCTASCTCBCGT                  GGBGCTBGACGACGSATACAATACCCACTAT
Reverse Fragments:                       TBCAGCGATBGAGSGCATTTGAAAGAGAGAGGAGACCSCGASCTGCTGCB
Assembled Construct: 401   GTCTTCTAACACAGASGTCGCTASCTCBCGTAAACTTTCTCTCTCCTCTGGBGCTBGACGACGSATACAATACCCACTAT 480

Forward Fragments:         GGTCSGGGAASGGGGSCCC                    GSGAGCGSACATSCGACTTTTCATGTATCTATAACGSTACG
Reverse Fragments:             BCCCTTBCCCCBGGGACAACTCCGTATAGTAGTTACBCTCGCBTGTABGC                  TGCBATGC
Assembled Construct: 481   GGTCSGGGAASGGGGSCCCTGTTGAGGCATATCATCAATGSGAGCGSACATSCGACTTTTCATGTATCTATAACGSTACG 560

Forward Fragments:         BCGTCCSAT                  CCGCBGSGATGAGGSAATAGTCGTGTTGTAGAGAACCBCTCABGGACBCG
Reverse Fragments:         SGCAGGBTATAATCGAGTGCTAGAATCGGCGSCBCTACTCCB                    GGSGAGTSCCTGSGCGGG
Assembled Construct: 561   BCGTCCSATATTAGCTCACGATCTTAGCCGCBGSGATGAGGSAATAGTCGTGTTGTAGAGAACCBCTCABGGACBCGCCC 640

Forward Fragments:                          SACCSGGGCSTBGCGTAGAAATGTTTTGCTTAAABASGCCTAGBGGGCBT
Reverse Fragments:         TATTCTGAAGAAGAACTBTGGBCCCGBASCGC                  TSTBCGGATCSCCCGSATCAATCGATGTTT
Assembled Construct: 641   ATAAGACTTCTTCTTGASACCSGGGCSTBGCGTAGAAATGTTTTGCTTAAABASGCCTAGBGGGCBTAGTTAGCTACAAA 720
Forward Fragments:              BCTTCCCSTGCCGABATTAGCGACTTAAGGATAACCGCGSABTGGASGGC                    SGSGG
Reverse Fragments:         TGACCSGAAGGGBACGGCTS                    GCGCBTSACCTBCCGGATTCTGTTTACGTGGCTGABCBCC
Assembled Construct: 721   ACTGGBCTTCCCSTGCCGABATTAGCGACTTAAGGATAACCGCGSABTGGASGGCCTAAGACAAATGCACCGACTSGSGG 800

Forward Fragments:         CTCTTSGGCSATTCATCTATAGAACTTGACBGGBGCGSGTASGGT                  GCCCBTTGSCGTBCCAG
Reverse Fragments:         GAGAABCCGB                  TGSCCSCGCBCATBCCATTATGTCTTCTATGCCGACGGGSAACBGCASGG
Assembled Construct: 801   CTCTTSGGCSATTCATCTATAGAACTTGACBGGBGCGSGTASGGTAATACAGAAGATACGGCTGCCCBTTGSCGTBCCAG 880

Forward Fragments:         TATCCATTCCATACGTTGGBAACCABTCCGGSG                    SGGAASCCGSCCGGBTATAGGTTTAGA
Reverse Fragments:                           CSTTGGTSAGGCCBCCGTAGTATACTTTCCCATATBCCTTBGGCBGGCCS
Assembled Construct: 881   TATCCATTCCATACGTTGGBAACCABTCCGGSGGCATCATATGAAAGGGTATASGGAASCCGSCCGGBTATAGGTTTAGA 960

Forward Fragments:         TGTTAGASTCGGTCSGCSAGSGT                  GGBTCGSGGAGTCCBAAATGGAATAGTAGAGCATCCGCG
Reverse Fragments:               TBAGCCAGBCGBTCBCAGTTACATTGTAACATGATCCSAGCBCCTCAGGS                    GCGC
Assembled Construct: 961   TGTTAGASTCGGTCSGCSAGSGTCAATGTAACATTGTACTAGGBTCGSGGAGTCCBAAATGGAATAGTAGAGCATCCGCG 1040

Forward Fragments:         BGSTCATTCSC                    SGTGCSACCCCACBGTGAAAAGTAGACGATCTAABTGTTGCBAGCGCSC
Reverse Fragments:         SCBAGTAAGBGATTTGTAAAGTGGTAGTATTBCACGBTGGGGTGSC
Assembled Construct: 1041  BGSTCATTCSCTAAACATTTCACCATCATAASGTGCSACCCCACBGTGAAAAGTAGACGATCTAABTGTTGCBAGCGCSC 1120

Forward Fragments:         T
Reverse Fragments:
Assembled Construct: 1121  T 1121
```

Figure S1: The 32A assembly. Shown (top two lines) are the forward and reverse oligonucleotide fragments (16 of each, respectively) together with their intended autonomous hybridization to give the target 1135 bp assembly (bottom line). The assembly was designed to be completed by filling in the gaps with Phusion DNA polymerase and sealing the nicks with *Taq* DNA ligase to give, before conversion, the AEGIS construct shown in the third line. Subsequently, **S** and **B** were converted to T and A, respectively, by conversion PCR.

```
Forward Fragments:                  AGCGGGATCCGACAGGGTTGACGATTACAAAGGCAGGAGGGCATC
Reverse Fragments:           TTCTCGGAGGGTCGATGGGGTTCGCATGTGTAGGTTCGCCCTAGGCTGTC     CCTCCCGTAG
Assembled Construct:  1      AAGAGCCTCCCAGCTACCCCAAGCGTACACATCCAAGCGGGATCCGACAGGGTTGACGATTACAAAGGCAGGAGGGCATC  80

Forward Fragments:           AACTG     TGAGGAGCTCACCGGATCAATACATGACGAAGTAGCCGATTTGGAGTGTT
Reverse Fragments:           TTGACAAAACAAAGAATTGGGTAGGACTCCTCGAGTGGCC     TCGGCTAAACCTCACAAAAACA
Assembled Construct:  81     AACTGTTTTGTTTCTTAACCCATCCTGAGGAGCTCACCGGATCAATACATGACGAAGTAGCCGATTTGGAGTGTTTTTGT  160

Forward Fragments:           ATGCCGAGGCGATAGCATTCTTTTTAAACACCTTTAGCCGAACTATGGCC
Reverse Fragments:           GTTTACATCTTAGTATACGGCTCCGCTATC     TCGGCTTGATACCGGTCTTCACTTCATAGT
Assembled Construct:  161    CAAATGTAGAATCATATGCCGAGGCGATAGCATTCTTTTTAAACACCTTTAGCCGAACTATGGCCAGAAGTGAAGTATCA  240

Forward Fragments:           AATCAGGCCTTGCACTTGCTACATTACTTTTCTAGACAAAGAGACGGGTT     CAAGT
Reverse Fragments:           TACCATTAGTCCGGAACGTG     TCTGTTTCTCTGCCCAATGAGCTCGATCAATACCAAAGTTCA
Assembled Construct:  241    ATGGTAATCAGGCCTTGCACTTGCTACATTACTTTTCTAGACAAAGAGACGGGTTACTCGAGCTAGTTATGGTTTCAAGT  320

Forward Fragments:           CCGTCATTGCTTGAAGGACCGAATTCATTAGCCGATAGGTACGTC     TAACGGCTCGTTCGT
Reverse Fragments:           GGCAGTAACG     CGGCTATCCATGCAGCAGAGTGCTCGAGATATCTTATTGCCGAGCAAGCA
Assembled Construct:  321    CCGTCATTGCTTGAAGGACCGAATTCATTAGCCGATAGGTACGTCGTCTCACGAGCTCTATAGAATAACGGCTCGTTCGT  400

Forward Fragments:           ATAACAATACACTTTCACACGTTCTTCAGTGACGT     CTGTCGCTCTTTGCGAAAGTAAGTT
Reverse Fragments:           TGCAAGAAGTCACTGCATCTCGAATAGGTGCAACGAAGACAGCGAGAAACGC
Assembled Construct:  401    ATAACAATACACTTTCACACGTTCTTCAGTGACGTAGAGCTTATCCACGTTGCTTCTGTCGCTCTTTGCGAAAGTAAGTT  480

Forward Fragments:           AACATGTTTGGACCACTGCCAGTAC     TAGCCAGGAAGAGCGATTACGGAAAGGTCAAAAAT
Reverse Fragments:           CTGGTGACGGTCATGCTCCTTATGGGTGTCTACTGATCGGTCCTTCTCGC     TA
Assembled Construct:  481    AACATGTTTGGACCACTGCCAGTACGAGGAATACCCACAGATGACTAGCCAGGAAGAGCGATTACGGAAAGGTCAAAAAT  560

Forward Fragments:           CTTTCCAGGGCACGT     CCGTAGGGATGACCCTCTAGAAGTCGAGGGGTAGTAGCTAGGCCA
Reverse Fragments:           GAAAGGTCCCGTGCAATAGTCGCCTTTTCTATTGTGGCATCCCTACTGGG     TCGATCCGGT
Assembled Construct:  561    CTTTCCAGGGCACGTTATCAGCGGAAAAGATAACACCGTAGGGATGACCCTCTAGAAGTCGAGGGGTAGTAGCTAGGCCA  640

Forward Fragments:           CAGAC     TCCAGACGTCCTGGTCTTAGAGAACATATGTAAACGACGTGTACCGTTCT
Reverse Fragments:           GTCTGACGAATCTCTGTATGAATCTAGGTCTGCAGGACCA     TGCTGCACATGGCAAGATGACC
Assembled Construct:  641    CAGACTGCTTAGAGACATACTTAGATCCAGACGTCCTGGTCTTAGAGAACATATGTAAACGACGTGTACCGTTCTACTGG  720

Forward Fragments:           ATTGGCCTCACGCTGATTGGTCTTATCAGACGCTGGGCGTTTAAACCGGT
Reverse Fragments:           CCTTACTCATTGATTTAACCGGAGTGCGAC     CCGCAAATTTGGCCACAACTCATCCGGAAA
Assembled Construct:  721    GGAATGAGTAACTAAATTGGCCTCACGCTGATTGGTCTTATCAGACGCTGGGCGTTTAAACCGGTGTTGAGTAGGCCTTT  800

Forward Fragments:           TGGCGTAAGTGGCGTGCTGAAGTCCTATAGTTTAGGAAGCAACAGCATGT     TGCGG
Reverse Fragments:           TAGCGACCGCATTCACCGCA     TCCTTCGTTGTCGTACAAAGTCCGCTTATGAGTTACAACGCC
Assembled Construct:  801    ATCGCTGGCGTAAGTGGCGTGCTGAAGTCCTATAGTTTAGGAAGCAACAGCATGTTTCAGGCGAATACTCAATGTTGCGG  880

Forward Fragments:           TTAACTGACGACAAGCATTACATTCACCATAAATGCCACAGGACG     GGCTGAGAAGGAAGC
Reverse Fragments:           AATTGACTGC     TTTACGGTGTCCTGCATGATACTTGATCTCAGCCTCCGACTCTTCCTTCG
Assembled Construct:  881    TTAACTGACGACAAGCATTACATTCACCATAAATGCCACAGGACGTACTATGAACTAGAGTCGGAGGCTGAGAAGGAAGC  960

Forward Fragments:           GGTATACTCTGTTTTCTTATAGTTCCGACCGACGT     AGCACACTTCCGCTCTCTTTCTGAG
Reverse Fragments:           TATCAAGGCTGGCTGCATATCCAACTGCAGTAATGATTCGTGTGAAGGCGAG
Assembled Construct:  961    GGTATACTCTGTTTTCTTATAGTTCCGACCGACGTATAGGTTGACGTCATTACTAAGCACACTTCCGCTCTCTTTCTGAG  1040

Forward Fragments:           TATGGTCCTTAAGACTGGGCACAAC     CGGTGGATCCAAGAGATTACACTGGCTTTACCCAA
Reverse Fragments:           TTCTGACCCGTGTTGTCTCTTCATCATGTGTCCACGCCACCTAGGTTCTC
Assembled Construct:  1041   TATGGTCCTTAAGACTGGGCACAACAGAGAAGTAGTACACAGGTGCGGTGGATCCAAGAGATTACACTGGCTTTACCCAA  1120

Forward Fragments:           GGATAGTACGCGAGT
Reverse Fragments:
Assembled Construct:  1056   GGATAGTACGCGAGT  1135
```

Figure S2: The 32B assembly. Shown (top two lines) are the forward and reverse synthetic fragments (16 of each) together with their intended overlap hybridization to give the target bp assembly (bottom line). The assembly was designed to be completed by filling in the gaps with Phusion DNA polymerase and sealing the nicks with T4 DNA ligase.

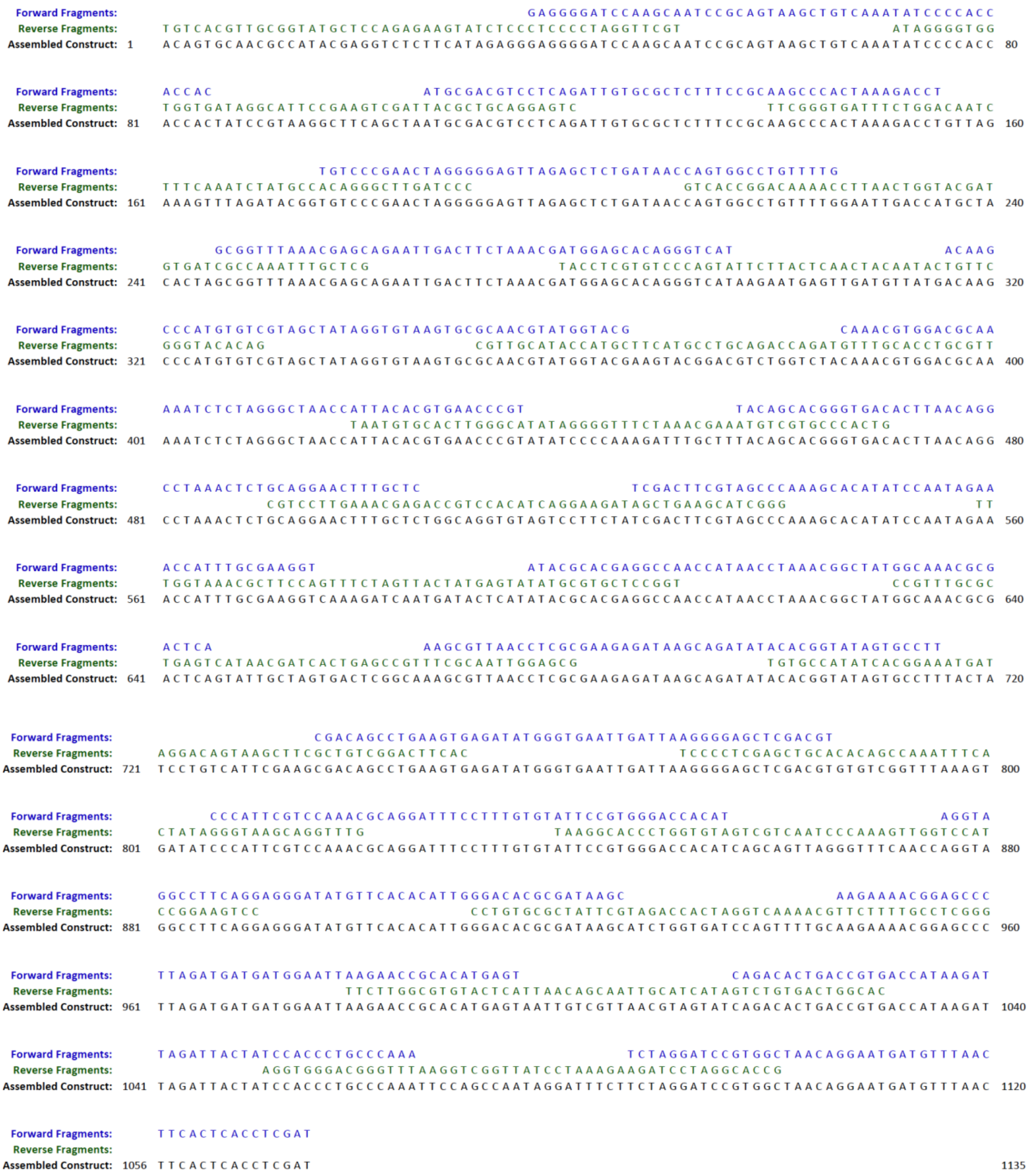

Figure S3: The 32C assembly. Shown (top two lines) are the forward and reverse oligonucleotide fragments (16 of each, respectively) together with their intended autonomous hybridization to give the target 1135 bp assembly (bottom line). The assembly was designed to be completed by filling in the gaps with Phusion DNA polymerase and sealing the nicks with T4 DNA ligase.

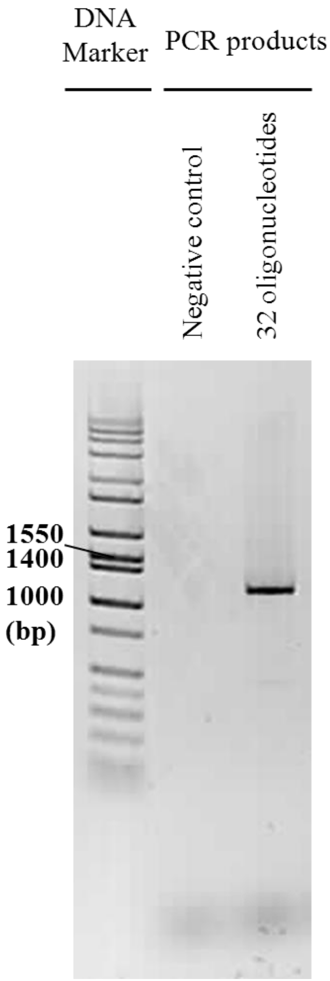

Figure S4: Two independent 16-fragments of the 32B construct were ligated by T4 DNA Ligase, and full-length assemblies were recovered by PCR. Shown is an agarose gel resolving the products, obtained after the second ligation, followed by PCR. Ladder is at left.

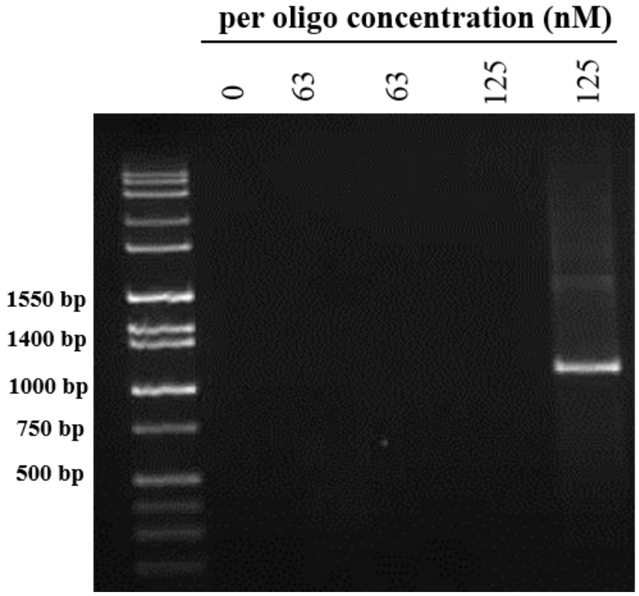

Figure S5: 32C assembly resolved on a 1.2% TAE agarose gel stained with ethidium bromide. Sample lanes from left to right represent 5 PCR amplifications (2 µl of each reaction): a non-template control PCR and two sets of duplicate AEL reactions with per-oligo concentrations of 63 and 125 nM, respectively. Ladder is at left. A band whose length (1135 bp) is consistent with the PCR product of the 32 oligonucleotide AEL construct is present in one, but not both replicates, of the two 32O AEL reactions at a per-oligo concentration of 125 nM. This amount is double that of 63 nM, the per-oligo concentration used in the AEL construction of the Kanamycin resistance gene as well as in two of the AEL reactions represented on the gel. The thermal profile was as follows: a pre-incubation (no enzyme) of 5 min at 80°C. 30 min at 40°C and 59 min at 50°C.

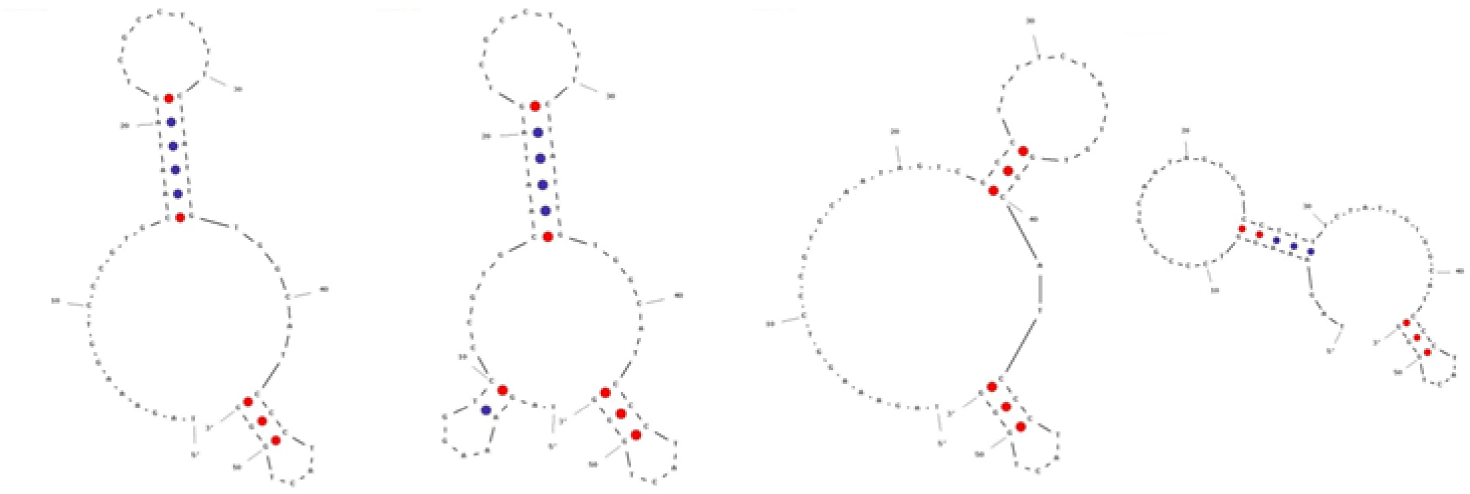

Figure S6: Predicted higher-order DNA structure of the Oligo #17 from the 32B assembly, obtained via Oligo Analyzer 3.1 (Integrated DNA Technologies). The four most possible hairpin structures are shown. Similar "folds" can be proposed, of course, for essentially any set of oligonucleotides built from only standard nucleotides.

References

1. Gibson, D. G., *Meth. Enzymol.* **2011,** *498*, 349-361.
2. Bradley, K. M., Benner, S. A. *Beilstein J. Org. Chem.* accompanying paper
3. Caruthers, M. H., Kleppe, K., van de Sande, J. H., Sgaramella, V., Agarwal, K.L., Büchi, H., Gupta, N.K., Kumar, A., Ohtsuka, E., RajBhandary, U.L., Terao, T., Weber, H., Yamada, T., Khorana, H.G. *J. Mol. Biol.* **1972**, *72*, 475-492.
4. Edge, M. D., Greene, A. R., Heathcliffe, G. R., Meacock, P. A., Schuch, W., Scanlon, D. B., Atkinson, T. C., Newton, C. R., Markham, A. F. *Nature* **1981,** *292*, 756-762.
5. Nambiar, K. P., Stackhouse, J., Stauffer, D. M., Kennedy, W. P., Eldredge, J. K., Benner, S. A. *Science* **1984,** *223*, 1299-1301.
6. Gibson, D. G., Benders, G.A., Andrews-Pfannkoch, C., *et al. Science* **2008,** *319*, 1215-1220.
7. Benner, S. A. *Acc. Chem. Res.* **2004**, *37*, 784-797
8. Benner, S. A., Yang, Z., Chen, F. *Comptes Rendus* **2011**, *14*, 372-387
